# Role of MXRA8 in Ross River Virus Disease Pathogenesis

**DOI:** 10.1128/mbio.00588-23

**Published:** 2023-04-10

**Authors:** Wern Hann Ng, Zheng L. Ling, Andrew J. Kueh, Marco J. Herold, Nicholas P. West, Nicholas J. C. King, Suresh Mahalingam, Xiang Liu

**Affiliations:** a Emerging Viruses, Inflammation and Therapeutics Group, Menzies Health Institute Queensland, Griffith University, Gold Coast, Queensland, Australia; b Global Virus Network (GVN) Centre of Excellence in Arboviruses, Griffith University, Gold Coast, Queensland, Australia; c School of Pharmacy and Medical Sciences, Griffith University, Gold Coast, Queensland, Australia; d Viral Immunopathology Laboratory, Infection, Immunity and Inflammation Research Theme, Charles Perkins Centre, School of Medical Sciences, Faculty of Medicine and Health, The University of Sydney, Sydney, New South Wales, Australia; e Sydney Institute for Infectious Diseases, Sydney Medical School, The University of Sydney, Sydney, New South Wales, Australia; f The Walter and Eliza Hall Institute of Medical Research, Melbourne, Victoria, Australia; g Department of Medical Biology, The University of Melbourne, Melbourne, Victoria, Australia; h Mucosal Immunology Group, Menzies Health Institute Queensland, Griffith University, Gold Coast, Queensland, Australia; Johns Hopkins Bloomberg School of Public Health

**Keywords:** MXRA8, Ross River virus, alphavirus, pathogenesis

## Abstract

Arthritogenic alphaviruses such as Ross River virus (RRV) and Chikungunya virus (CHIKV) are responsible for large-scale epidemics that cause debilitating acute and chronic musculoskeletal diseases. MXRA8 was recently discovered as an entry receptor for multiple alphaviruses including CHIKV, RRV, Mayaro virus (MAYV), and O’nyong-nyong virus (ONNV). However, the role of MXRA8 in the development of alphavirus-induced musculoskeletal inflammation has not yet been fully studied. Here, we attempt to fully characterize the contribution of MXRA8 to RRV disease in an established mouse model. MXRA8 knockout (MXRA8^−/−^) mice generated on a C57BL/6J background, showed abrogated disease signs and reduced viral replication, which correlated with lower viral load, diminished proinflammatory cytokines, and limited cell infiltrates in inflamed tissues. Immunomodulation genes were upregulated to higher levels in RRV-infected wild-type (WT) mice than in MXRA8^−/−^ mice. Intriguingly, *Cdkn1a* and *Ifi44* genes in blood and CD127/IL7RA, CD45, BatF3, IFNGR, Ly6G/Ly6C, CD40, CD127, F4/80, and MHC-II genes in quadriceps were found to be upregulated in RRV-infected MXRA8^−/−^ mice compared to WT mice. Our results showed an essential role of MXRA8 in the immune response of mice infected with RRV and, more importantly, demonstrated novel changes in immunomodulation genes, which shed light on the immunopathogenesis of alphavirus-induced disease.

## INTRODUCTION

Arthritogenic alphaviruses such as Ross River virus (RRV), Chikungunya virus (CHIKV), Mayaro virus (MAYV), and O’nyong-nyong virus (ONNV) are positive-sense single-stranded RNA viruses transmitted by mosquitoes (1). These alphaviruses are emerging pathogens that have been progressively expanding their global distribution (2). Symptomatic infections of alphaviruses are frequently linked to musculoskeletal diseases such as arthralgia, arthritis, and myalgia which can be chronic and debilitating (3–5). There are currently no specific treatments or vaccines available for arthritogenic alphaviruses (2, 6).

A range of cell surface molecules have been shown to facilitate alphavirus attachment to host cells, including heparan sulfate, C-type lectins, and phosphatidylserine (7). However, the major entry receptor mediating alphavirus internalization was not identified until recently. A genome-wide CRISPR-Cas9-based screening in mouse cells identified MXRA8 as the main entry receptor for multiple arthritogenic alphaviruses, including RRV, CHIKV, MAYV, and ONNV. MXRA8 (also known as DICAM, ASP3, or limitrin) is a conserved cell surface adhesion molecule found in mammals, birds, and amphibians. MXRA8 is mainly expressed on osteogenic, epithelial, myeloid, and mesenchymal cells and is distributed in high concentrations in the plasma membrane (8–11). Mechanistically, MXRA8 interacts with integrin β3 and is a known negative regulator of osteoclast differentiation (9). Knockdown of MXRA8 in mice increases the expression of osteoclast-associated Ig-like receptor (OSCAR) and integrin β3, suggesting that MXRA8 regulates osteoclast differentiation negatively by suppressing integrin β3 expression. In an experimental autoimmune optic neuritis (EAON) and colitis model, mice deficient in MXRA8 exhibited more severe inflammation, compared to wild-type (WT) mice (12, 13). Conversely, patients diagnosed with optic neuritis were also found to express higher levels of MXRA8 compared to healthy patients (12). MXRA8 mRNA and protein were found to increase gradually during the onset of osteoclastogenesis until integrin β3 reached its peak but reverted to normal expression levels in the later stages, which is associated with reduced levels of integrin β3. In Caco-2 cells, MXRA8 RNA and protein levels were also shown to increase significantly when treated with tumor necrosis factor alpha (TNF-α) and/or gamma interferon (IFN-γ) in a study by Han et al. (13). These previous studies suggest MXRA8, as well as acting as an alphavirus entry receptor, may also modulate the pathogenesis of alphavirus-induced arthritis. While the role of MXRA8 in CHIKV infection has been characterized in previous studies (14, 15), more details of the function of MXRA8 in other alphaviral disease pathogenesis are needed to better understand alphavirus-host interactions. Here, by utilizing an established RRV mouse model, we examined RRV disease development, leukocyte infiltration, and antiviral and proinflammatory cytokine production in a MXRA8-deficient mouse model. Observations from this study provide further insight into the role of MXRA8 in alphaviral disease and shed light on potential therapeutic strategies.

## RESULTS

### Impact of MXRA8 in RRV-induced disease in mouse.

**FIG 1:**
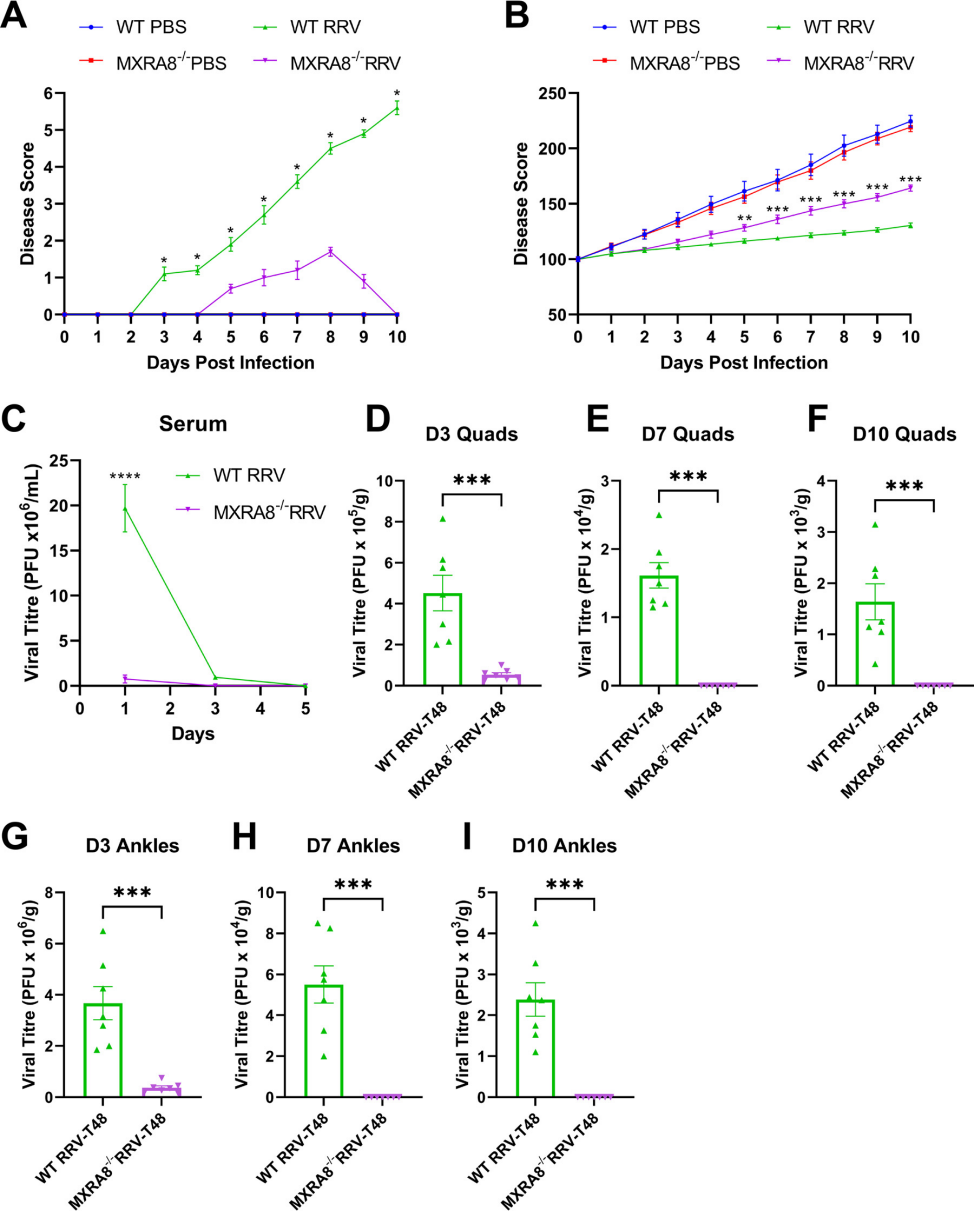
MXRA8 is required for optimal RRV disease development and viral replication. (A and B) WT and MXRA8^−/−^ mice (*n* = 7 per group) were infected with RRV at 10^4^ PFU or mock infected with PBS. Mouse disease and weight were monitored daily up to 10 dpi. The data are representative of two independent experiments. *, *P < *0.05; **, *P < *0.01; ***, *P < *0.001 (using two-way ANOVA with Bonferroni posttest). (C) Mouse sera of infected WT and MXRA8^−/−^ mice were collected at 1, 3, and 5 dpi and processed for plaque assay. The data, representative of two independent experiments, are presented as means ± the standard errors of the mean (SEM). ****, *P < *0.0001 (using two-way ANOVA with the Bonferroni posttest). (D to I) Quadriceps and ankles of the infected WT and MXRA8^−/−^ mice were harvested at 3, 7, and 10 dpi. Viral titers in these tissues were determined by plaque assay. Dots represent individual animals (*n* = 7). The data, representative of two independent experiments, are presented as means ± the SEM. ***, *P < *0.001 (using a *t* test with Mann-Whitney posttest).

**FIG 2:**
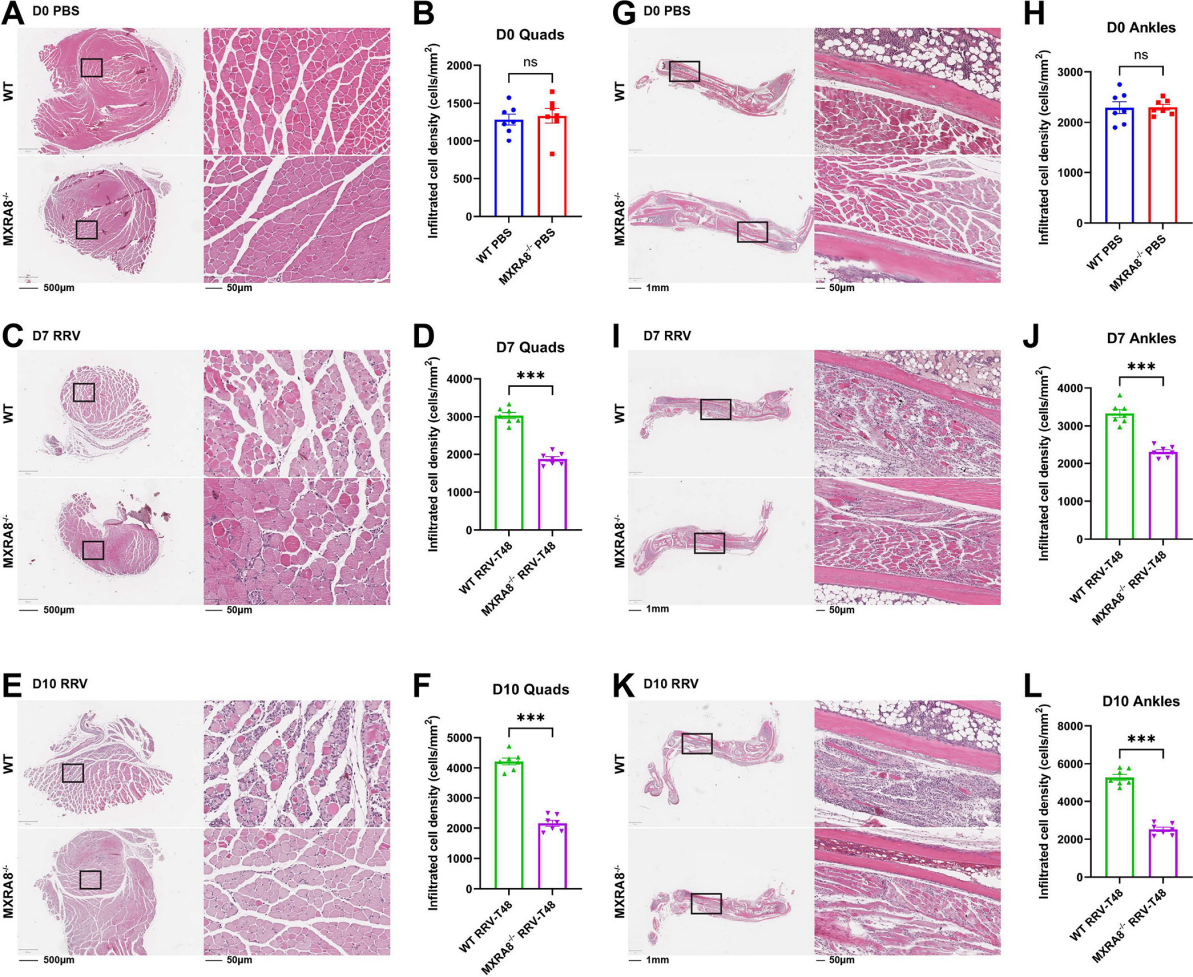
MXRA8 facilitates cellular infiltration in the quadriceps and ankles in mice with RRV disease. WT and MXRA8^−/−^ mice (*n* = 7 per group) were infected with RRV at 10^4^ PFU. Quadriceps (A, C, and E) and ankle joints (G, I, and K) were collected at 0, 7, and 10 dpi and processed for H&E staining. Microscopy images shown are representative of *n* = 7 mice per group. The numbers of infiltrated cells were analyzed using QuPath. (B, D, F, H, J, and L) Dots represent individual animals (*n* = 7). The data, representative of two independent experiments, are presented as means ± the SEM. ***, *P < *0.001 (using a *t* test with the Mann-Whitney posttest; ns, not significant).

To corroborate the role of MXRA8 in RRV disease (RRVD) progression, we used an established mouse model as described previously (16, 17). Wild-type C57BL/6J (WT) and MXRA8^−/−^ mice were infected subcutaneously with 10^4^ PFU of RRV or mock-infected with phosphate-buffered saline (PBS). Mice were scored daily for clinical signs. RRV-infected WT mice developed more severe RRVD, as shown by the onset of hind limb dysfunction at 6 or 7 days postinfection (dpi), whereas MXRA8^−/−^ mice experienced lethargy only at peak disease and did not experience hind limb dysfunction throughout the 10-day period (Fig. 1A). At 10 dpi, WT mice showed severe motor impairment and were unable to walk or stand on their hind legs, whereas MXRA8^−/−^ mice showed complete remission of RRVD. Consistently, RRV-infected MXRA8^−/−^ mice showed greater mass gain compared to RRV-infected WT mice (Fig. 1B). To assess whether the reduced clinical signs of RRV-infected MXRA8^−/−^ mice were associated with viral load, the virus titers in serum (Fig. 1C), the quadriceps (Fig. 1D to F), and the ankles (Fig. 1G to I) were determined at the indicated time points following infection. RRV titers in the sera of WT mice (~19.7 × 10^6^ PFU/mL) were significantly higher at 1 dpi, compared to MXRA8^−/−^ mice (~0.75 × 10^6^ PFU/mL), but by 3 dpi, RRV titers in the sera of WT mice (~0.94 × 10^6^ PFU/mL) were only slightly higher than in MXRA8^−/−^ mice (~0.007 × 10^6^ PFU/mL) and not statistically significant. By 5 dpi, virus titers in both WT and MXRA8^−/−^ sera were below the detection limit. This indicates that MXRA8^−/−^ mice have reduced viral replication compared to WT mice. Similarly, RRV titers in the quadriceps muscle and ankles of MXRA8^−/−^ mice were lower at all time points (3, 7, and 10 dpi) than in WT mice. To further determine the role of MXRA8 in RRV infection within an animal model, we administered anti-MXRA8-blocking monoclonal antibodies (MAbs) via an intraperitoneal route to WT mice at 6 h preinfection and 8 and 16 h postinfection. Serum and spleen, quadriceps muscle, and ankle joint tissues were collected, and virus titers were determined by plaque assay. RRV titers in all tissues collected from mice administered with anti-MXRA8 were significantly reduced compared to WT mice, suggesting that MXRA8 may act as therapeutic target in RRV disease (see Fig. S2 in the supplemental material). Inflammatory cell infiltration and tissue damage in the ankles and quadriceps, which are inflamed during RRV disease, were examined, using hematoxylin and eosin (H&E) staining (Fig. 2A, C, E, G, I, and K). In PBS mock-infected mice, tissue damage and cell infiltrates were comparable between WT and MXRA8^−/−^ mice (Fig. 2B and H). More severe tissue damage and marked cell infiltration were observed in WT mice at 7 and 10 dpi in quadriceps compared to the MXRA8^−/−^ mice (Fig. 2C to F and I to L). These results agree with findings from Zhang et al. (15) that MXRA8 is crucial for optimal RRV infection and disease development.

10.1128/mbio.00588-23.2 FIG S2 Administration of anti-MXRA8 MAbs prevents optimal RRV replication *in vivo*. WT mice were administered with anti-MXRA8 MAbs or antibody vehicle intraperitoneally at −6 h preinfection and at 8 and 16 h postinfection with 10^4^ PFU of RRV per animal. Sera (A), spleens (B), quadriceps (C), and ankles (D) were harvested at 1 dpi. The viral titers in these tissues were determined by plaque assay. Dots represent individual animals (*n* = 3). The data are presented as means ± the SEM. *, *P* < 0.05; **, *P* < 0.01 (using an unpaired *t* test). Download FIG S2, PDF file, 1.1 MB. Copyright © 2023 Ng et al. 2023 Ng et al. https://creativecommons.org/licenses/by/4.0/ This content is distributed under the terms of the Creative Commons Attribution 4.0 International license.

### Cartilage thickness and osteoclastogenic activity in RRVD is modulated by MXRA8.

**FIG 3:**
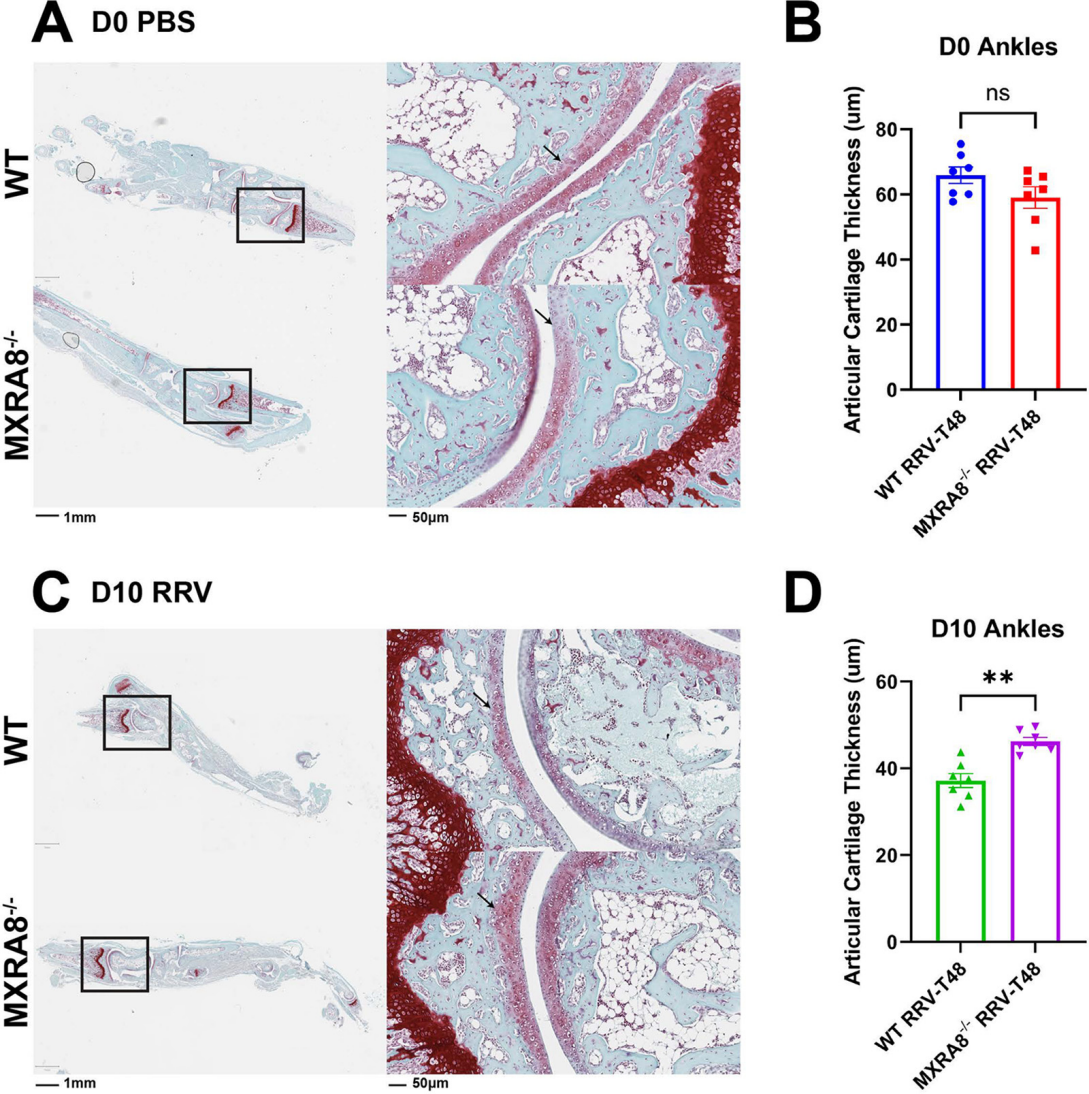
MXRA8 is associated with articular cartilage damage in the ankles of mice with RRV disease. WT and MXRA8^−/−^ mice (*n* = 7 per group) were infected with RRV at 10^4^ PFU. Ankles were collected from these infected mice at 0 dpi (A and B) and 10 dpi (C and D) and processed for Safranin-O staining. The thickness of articular cartilage was measured using ImageScope (the black arrows indicate the region measured). Dots represent individual animals (*n* = 7). The data, representative of two independent experiments, are presented as means ± the SEM. **, *P < *0.01 (using a *t* test with the Mann-Whitney posttest; ns, not significant).

RRV infection is known to cause pathological bone loss and cartilage degradation (18, 19). To assess whether MXRA8 plays a role in pathological cartilage/bone erosion in RRVD, the articular damage of MXRA8^−/−^ mice and WT mice following RRV infection was determined. Ankle joints were harvested from RRV- or mock-infected MXRA8^−/−^ and WT mice, and cartilage samples were stained with Safranin-O staining to assess articular cartilage damage. The articular cartilage thickness was slightly thinner in mock-infected MXRA8^−/−^ mice (~59.06 μm) compared to mock-infected WT mice (~65.88 μm), but this was not statistically significant (Fig. 3A and B). On the other hand, the thickness of cartilage in RRV-infected WT mice (~37.18 μm) was shown to be significantly thinner compared to RRV-infected MXRA8^−/−^ mice (~46.2 μm) at 10 dpi (Fig. 3C and D), indicating greater cartilage damage in RRV-infected WT mice.

Next, we compared the osteoclastogenic activity between RRV-infected WT mice and MXRA8^−/−^ mice. Knee tissues were harvested at 10 dpi and stained for osteoclasts (tartrate-resistant acid phosphatase [TRAP] cells) (see Fig. S3). Notably, numbers of osteoclasts (TRAP-positive cells) were elevated at baseline (mock-infected mice) in MXRA8^−/−^ mice compared to WT mice, suggesting that there is a higher level of osteoclastogenic activity in MXRA8-deficient mice at baseline than in WT mice (see Fig. S3A, B, and E). However, both RRV-infected WT and MXRA8^−/−^ groups displayed increased numbers of osteoclasts compared to their respective mock-infected groups (see Fig. S3A to E). While the numbers of osteoclasts in RRV-infected MXRA8^−/−^ and RRV-infected WT mice were comparable (see Fig. S3C, D, and E), this nevertheless indicates the relative RRVD-induced increase in osteoclastogenic activity in MXRA8^−/−^ mice was lower than in WT mice and supports our previous observation of RRVD-induced bone loss.

10.1128/mbio.00588-23.3 FIG S3 Osteoclastogenic activities are not regulated by MXRA8^−/−^ during RRV infection. WT and MXRA8^−/−^ mice were infected with RRV at 10^4^ PFU or mock infected with PBS. (A to D) Knees and ankles from the infected mice were collected at 10 dpi and processed for TRAP staining. TRAP positive cells were indicated with black arrows. Images are representative of the results for seven mice per group from two independent experiments. Dotted squares indicate ROIs. (E) The numbers of TRAP-positive cells were analyzed using ImageJ. *, *P* < 0.05; ****, *P* < 0.0001 (using one-way ANOVA, with a Brown-Forsythe posttest). Download FIG S3, PDF file, 1.0 MB. Copyright © 2023 Ng et al. 2023 Ng et al. https://creativecommons.org/licenses/by/4.0/ This content is distributed under the terms of the Creative Commons Attribution 4.0 International license.

### Reduced immune cell infiltration and proinflammatory cytokines and chemokines in MXRA8^–/–^ mice.

**FIG 4:**
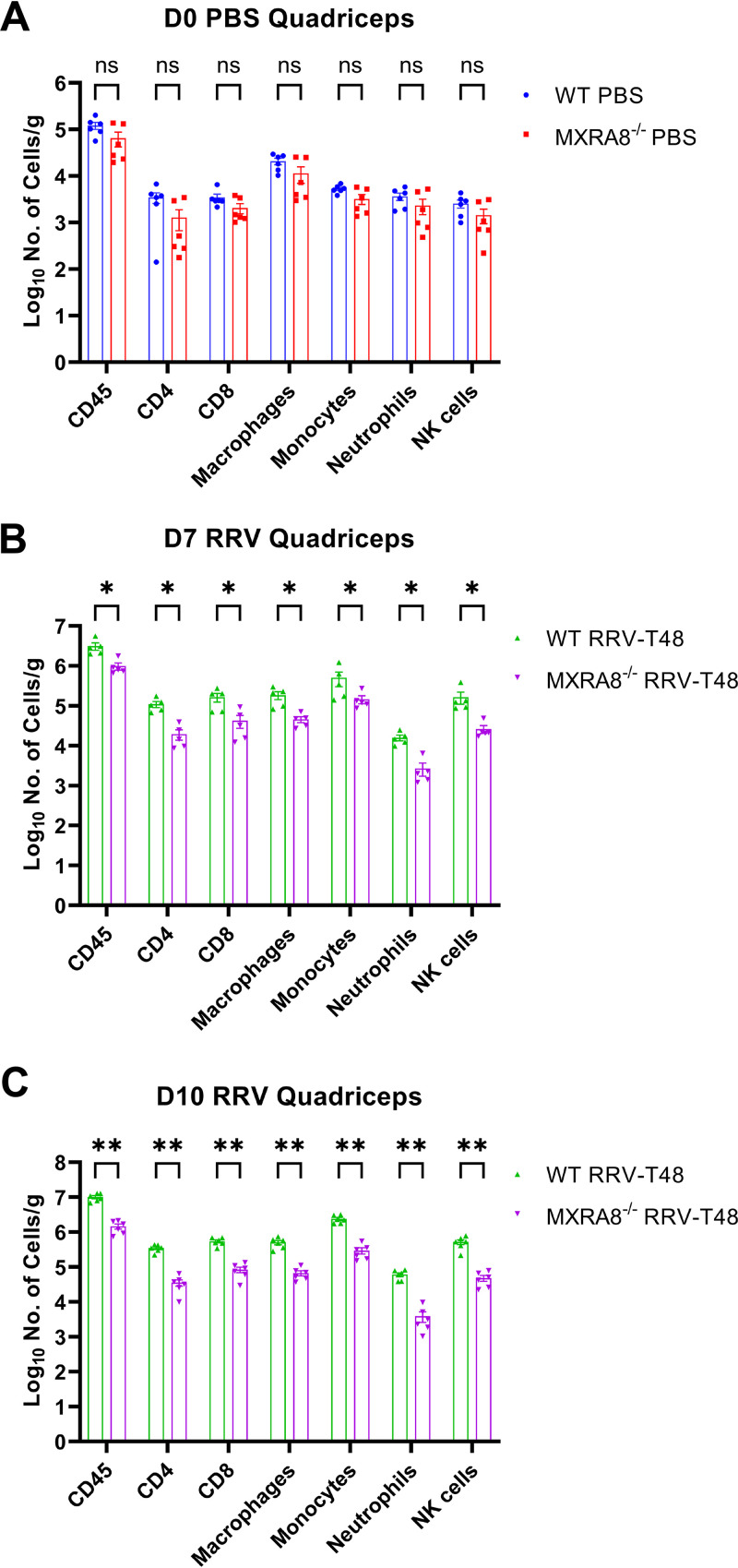
Absence of MXRA8 is associated with reduction in immune cell population in RRV-infected mice quadriceps. WT and MXRA8^−/−^ mice (*n* = 7 per group) were infected with RRV at 10^4^ PFU. Quadriceps were harvested from the infected mice at 0 dpi (A), 7 dpi (B), and 10 dpi (C). Live singlet cells were gated for CD45^+^ and subsequently excluded from NK^+^ cell gate. CD3^+^ cells were gated for CD4^+^ and CD8^+^ cells. CD3^−^ cells were gated first for neutrophils (Ly6G^+^) and then for macrophages (CD11b^+^, Ly6G^–^, and Ly6C^low^) and inflammatory monocytes (CD11b^+^, Ly6G^–^, and Ly6C^high^). Dots represent individual animals (*n* = 5 to 6). The data, representative of two independent experiments, are presented as means ± the SEM. *, *P < *0.05; **, *P < *0.01 (using a *t* test with the Mann-Whitney posttest; ns, not significant).

Having demonstrated that the absence of MXRA8 gene in mice showed reduced viral replication, we next investigated its impact in leukocyte recruitment in infected mice using flow cytometry. Since the quadriceps muscles are key target tissues for RRV infection, we measured leukocyte infiltration in these tissues at 7 and 10 dpi, i.e., the peak of RRVD. Total CD45^+^ leukocyte numbers and all defined leukocyte subsets in mock-infected MXRA8^−/−^ mice were consistently lower than in mock-infected WT mice, although this was not statistically significant (Fig. 4A). Infiltrating leukocyte subsets, including CD4^+^ T cells, CD8^+^ T cells, macrophages (CD11b^+^ Ly6C^low^ Ly6G^–^), inflammatory monocytes (CD11b^+^ Ly6C^hi^ Ly6G^–^), neutrophils (Ly6G^+^), and NK cells (NK1.1^+^) were all significantly lower in RRV-infected MXRA8^−/−^ mice at 7 and 10 dpi than in RRV-infected WT mice at these time points (Fig. 4B and C).

Mass cytometry data of draining lymph nodes (DLNs) and spleens were processed by Uniform Manifold Approximation and Projection (UMAP) for dimension reduction and visualization. Clusters of major cell types were identified on the UMAP plots, based on the expression of fundamental cell markers, such as CD3, CD4, CD8, CD11b, CD19, etc. Positional shifts on the UMAP plots represent phenotypic changes in these populations (see Fig. S4 and 5). CCR7, Ly6C, and CD11b were major contributors to phenotype changes in lymphocytes and CCR2, Ly6C, CD163, and CD64 contributed to the phenotype shifts observed in myeloid cells (see Fig. S4 and 5).

10.1128/mbio.00588-23.4 FIG S4 MXRA8^−/−^ mice tend to have delayed immune response with reduced early immune cell activation in DLNs. MXRA8^−/−^ and WT mice were infected with 10^4^ PFU of RRV-T48. The popliteal DLNs were harvested from mock-infected and day 3 and 7 postinfected mice and processed for mass cytometry and analyzed by UMAP for dimension reduction. The identities of basic clusters were determined and gated based on the expression of conventional immune cell markers, such as CD3, CD4, CD8, CD19, CD11b, CD11c, Ly6G, NK1.1, Siglec F, and TCR γδ. Cell phenotype changes are identified as position shifts on UMAP plots (A and H). CCR7, Ly6C, and CD11b were identified to be major contributors to phenotype shifts in lymphocytes (B, C, D, E, F, and G), while CCR2, Ly6C, CD163, and CD64 contributed to phenotype shifts in myeloid cells on UMAP plots (I, J, K, L, and M). The data are presented as means ± the SEM. *, *P* < 0.05; **, *P* < 0.01; ***, *P* < 0.001; ****, *P* < 0.0001 (using two-way ANOVA with a Holm-Šídák posttest; ns, not significant). Download FIG S4, PDF file, 1.1 MB. Copyright © 2023 Ng et al. 2023 Ng et al. https://creativecommons.org/licenses/by/4.0/ This content is distributed under the terms of the Creative Commons Attribution 4.0 International license.

In both DLNs and the spleen, these surface antigens were found to be increased on T and B lymphocytes, as well as CD11b^+^ myeloid cells in both RRV-infected MXRA8^−/−^ and WT mice. However, the levels of CCR7, Ly6C, CD11b, CCR2, CD163, and CD64 tended to be lower at 3 dpi and higher at 7 dpi in MXRA8^−/−^ mice compared to WT mice (see Fig. S4 and 5), suggesting a delayed induction of these cell markers in MXRA8^−/−^ mice. Furthermore, the peak of these molecules in MXRA8^−/−^ mice at 7 dpi tended to be lower than the peak in WT mice at 3 dpi. There were also differences in the frequencies of individual cell populations, the most obvious being the differential frequencies of B cells, CD4^+^ and CD8^+^ T cells in both DLNs and the spleen (see Fig. S6). The kinetics of the population of these lymphocytes highlighted their crucial roles in RRV disease pathogenesis.

10.1128/mbio.00588-23.5 FIG S5 MXRA8^−/−^ mice tend to have delayed immune response with reduced early immune cell activation in spleen. MXRA8^−/−^ and WT mice were infected with 10^4^ PFU of RRV-T48. The spleens were harvested from mock-infected and day 3 and 7 postinfected mice and processed for mass cytometry and analyzed by UMAP for dimension reduction. The identities of basic clusters were determined and gated based on the expression of conventional immune cell markers, such as CD3, CD4, CD8, CD19, CD11b, CD11c, Ly6G, NK1.1, Siglec F, and TCR γδ. Cell phenotype changes are identified as position shifts on UMAP plots (A and G). CCR7, Ly6C, and CD11b were identified to be major contributors to phenotype shifts in lymphocytes (B, C, D, E, and F), while CCR2, Ly6C, CD163, and CD64 contributed to phenotype shifts in myeloid cells on UMAP plots (H, I, J, K, L, and M). The data are presented as means ± the SEM. *, *P* < 0.05; **, *P* < 0.01; ***, *P* < 0.001; ****, *P* < 0.0001 (using two-way ANOVA with a Holm-Šídák posttest; ns, not significant). Download FIG S5, PDF file, 1.1 MB. Copyright © 2023 Ng et al. 2023 Ng et al. https://creativecommons.org/licenses/by/4.0/ This content is distributed under the terms of the Creative Commons Attribution 4.0 International license.

10.1128/mbio.00588-23.6 FIG S6 MXRA8^−/−^ mice have differential frequencies during anti-viral immune activation. Frequencies of the indicated subsets harvested from the popliteal DLNs (A) and spleens (B) harvested from mock infected and day 3 and 7 postinfected mice were calculated as a percentage of the CD45^+^ population from MXRA8^−/−^ and WT mice infected with 10^4^ PFU of RRV. The data are presented as means ± the SEM. *, *P* < 0.05; **, *P* < 0.01; ***, *P* < 0.001; ****, *P* < 0.0001 (using two-way ANOVA with a Holm-Šídák posttest; ns, not significant). Download FIG S6, PDF file, 0.2 MB. Copyright © 2023 Ng et al. 2023 Ng et al. https://creativecommons.org/licenses/by/4.0/ This content is distributed under the terms of the Creative Commons Attribution 4.0 International license.

**FIG 5:**
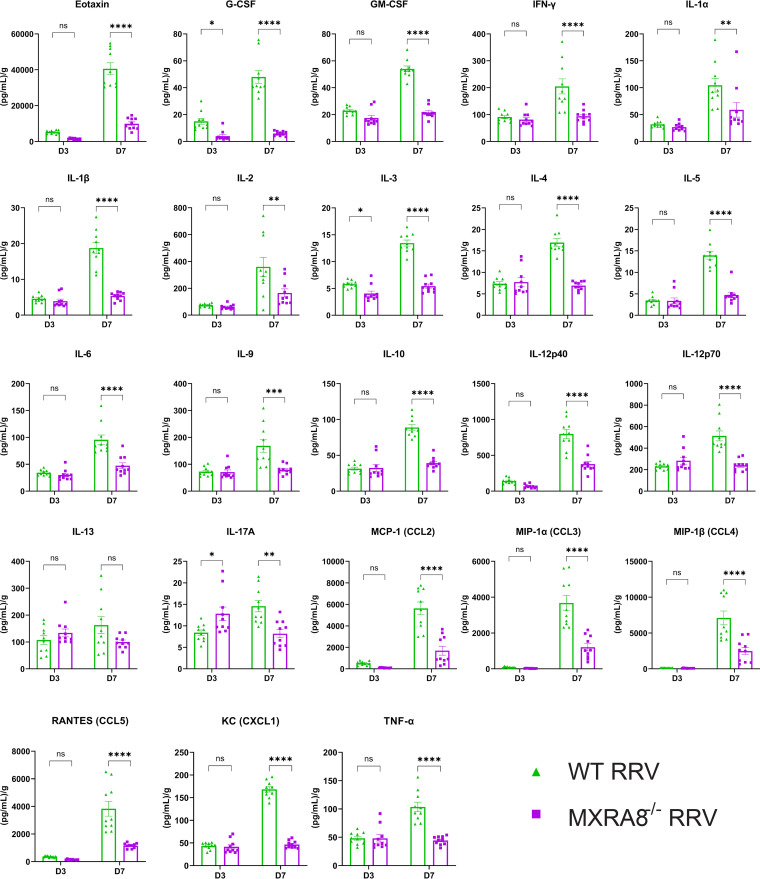
MXRA8 is associated with proinflammatory cytokine and chemokines in mice with RRV disease. WT and MXRA8^−/−^ mice were infected with RRV at 10^4^ PFU. Quadriceps were collected at 3 and 7 dpi and processed for cytokine/chemokine multiplex assay. Dots represent individual animals (*n* = 10). The data are presented as means ± the SEM. *, *P < *0.05; **, *P < *0.01; ***, *P < *0.001; ****, *P < *0.0001 (using two-way ANOVA with the Holm-Šídák posttest; ns, not significant).

We further profiled cytokines and chemokines in the mouse quadriceps muscles in RRV-infected WT and MXRA8^−/−^ mice at 3 and 7 dpi. In line with the leukocyte data, the proinflammatory cytokines and chemokines, Eotaxin, G-CSF, GM-CSF, IFN-γ, interleukin-1α (IL-1α), IL-1β, IL-2, IL-3, IL-4, IL-5, IL-6, IL-9, IL-10, IL-12, CCL2, CCL3, CCL4, CCL5, CXCL1, and TNF-α were all at significantly lower levels at 7 dpi in the quadriceps of MXRA8^−/−^ mice (Fig. 5). Interestingly, however, IL-17A was shown to be higher at 3 dpi in MXRA8^−/−^ mice compared to WT mice, suggesting the deficiency of MXRA8 may potentially alter T cell differentiation and Th17 cell recruitment in the early stages of RRVD. Taken together, consistent with the reduced disease signs observed, this indicates that RRV does not induce severe inflammation in MXRA8-deficient mice.

### MXRA8 alters the immune transcriptional profile of mice during RRVD.

**FIG 6:**
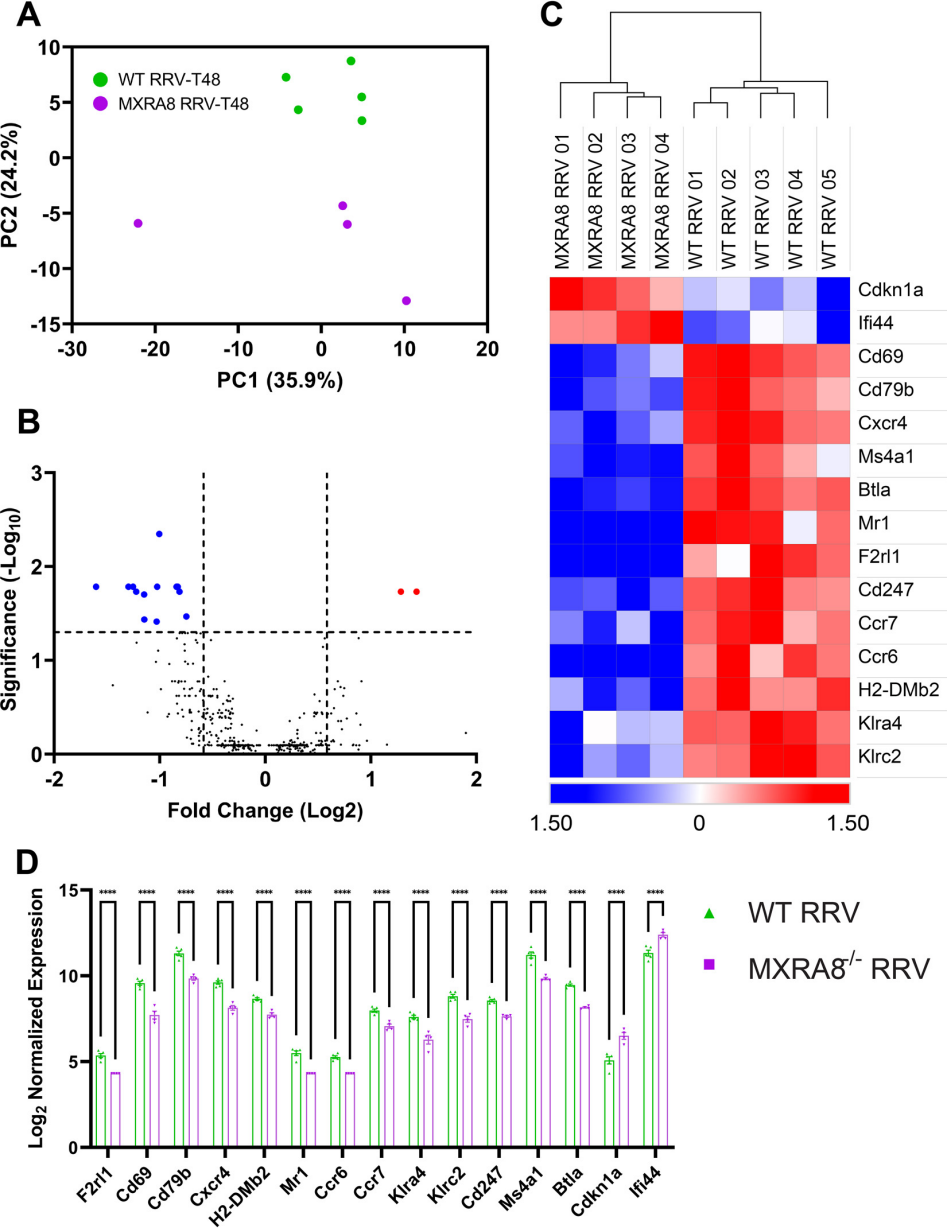
Cdkn1a and Ifi44 were upregulated in the blood of RRV-infected MXRA8^−/−^ mice. WT and MXRA8^−/−^ mice were infected with RRV at 10^4^ PFU. Sera were harvested from infected mice at 7 dpi. (A) PCA clustering between WT and MXRA8^−/−^ RRV-infected mice. (B and C) Volcano plot (B) and heatmap (C) of gene expression data. The data are normalized to hypoxanthine phosphoribosyltransferase (HRPT), and the differential expression is shown as the Z-score. Red dots indicate significantly increased expression of genes, and blue dots indicate significantly decreased expression of genes. (D) Comparison of genes, plotted as a bar graph. Dots represent individual animals (*n* = 4 to 5). The data are presented as means ± the SEM. *, *P < *0.05; **, *P < *0.01; ***, *P < *0.001; ****, *P < *0.0001 (using two-way ANOVA with the Šídák posttest; ns, not significant).

To corroborate the effect of MXRA8 on immune response at a transcriptional level, blood was isolated from RRV-infected mice at 7 dpi and differential gene expression analysis was conducted using NanoString. Principal-component analysis (PCA) clearly segregated RRV-infected WT and MXRA8^−/−^ mice, based on gene expression (Fig. 6A). Among 770 genes examined, 15 genes were differentially expressed (Q < 0.05) in the serum of RRV-infected MXRA8^−/−^ mice compared to WT mice. MXRA8-deficient mice upregulated Cdkn1a and Ifi44, whereas the other 13 genes, including CD69, CXCR4, MS4A1, CD79b, MR1, F2RL1, CD247, BTLA, CCR6, CCR7, H2-DMb2, KLRA4, and KLRC2, were downregulated (Fig. 6B to D).

**FIG 7:**
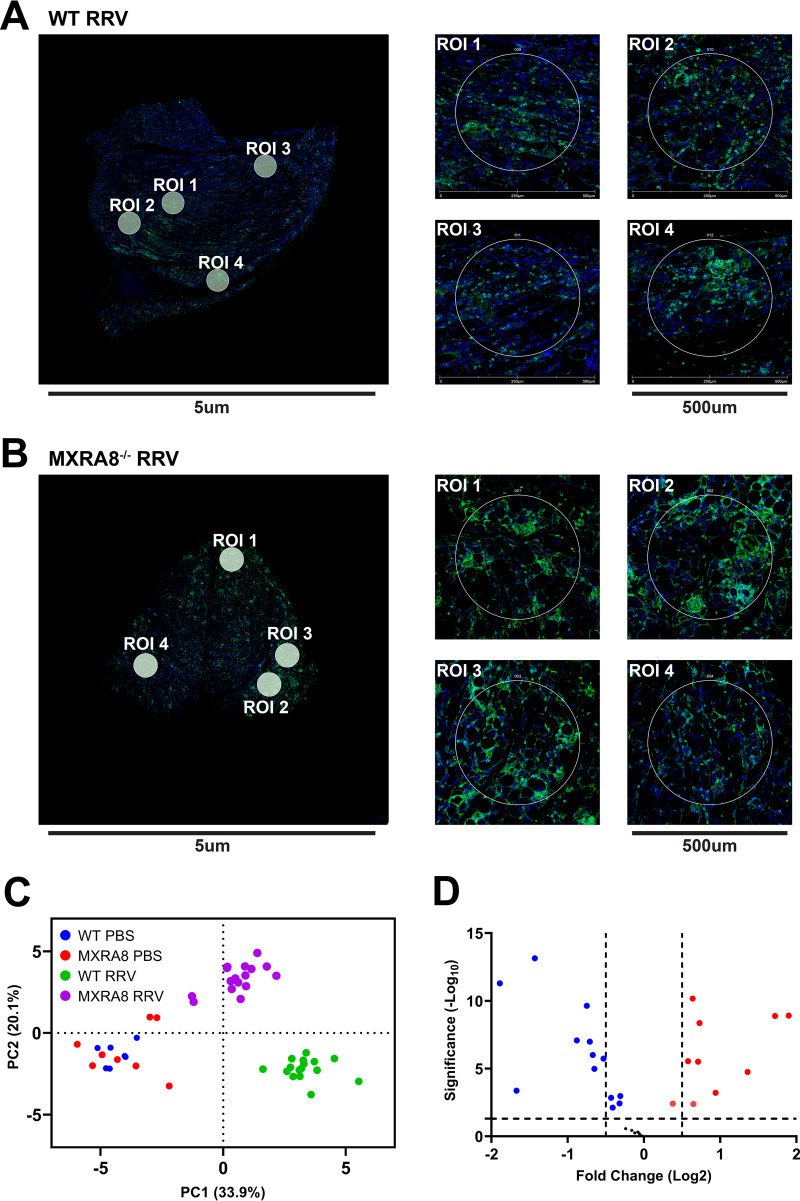
Immune transcriptional profile of RRV-infected MXRA8^−/−^ mice. WT and MXRA8^−/−^ mice were infected with RRV at 10^4^ PFU or mock infected with PBS. Quadriceps were harvested from infected mice at 10 dpi. Gene transcription profiles were analyzed using R. (A and B) Representative images of WT (A) and MXRA8^−/−^ RRV-infected (B) mice (*n* = 4 mice per group; two ROIs were selected from the mock-infected group [data not shown], and four ROIs were selected from the RRV-infected group). (C) PCA clustering between WT and MXRA8^−/−^ mock and RRV-infected mice. (D) Volcano plot of gene expression data. Red dots indicate significant increases in the fold change of genes, and blue dots indicates significant decreases in the fold change of genes. The samples are normalized to a WT control group.

**FIG 8:**
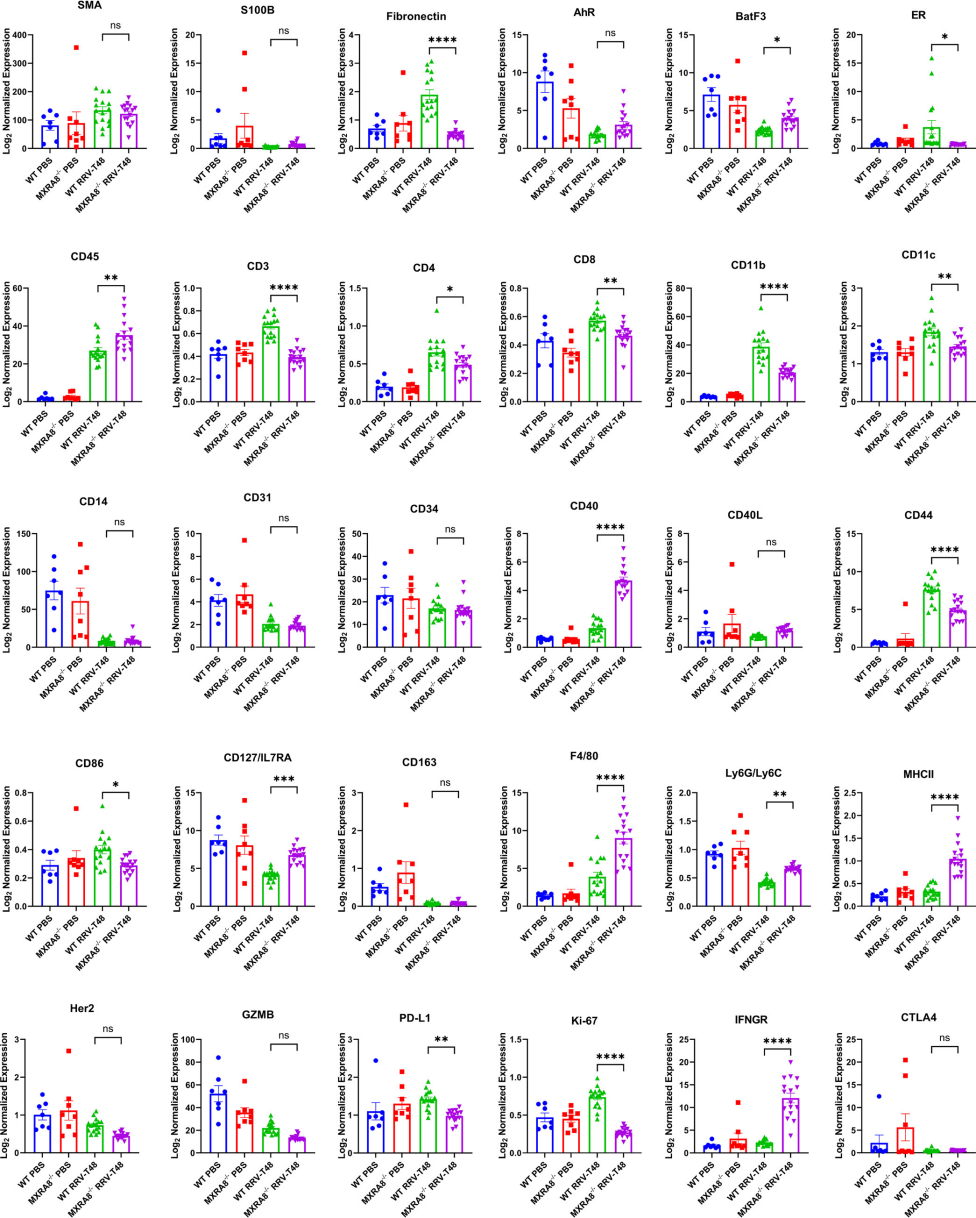
DSP spatial profile of RRV-infected WT and MXRA8^−/−^ mice. WT and MXRA8^−/−^ mice were infected with RRV at 10^4^ PFU or mock-infected with PBS. Quadriceps were harvested from WT and MXRA8^−/−^ mock- or RRV-infected mice at 10 dpi. Gene transcription profiles were analyzed using R. Differences in gene expression were plotted out in bar graphs. The data are normalized to a WT control group. Dots represents individual ROIs (*n* = 7, 8, or 16). The data are presented as means ± the SEM. *, *P < *0.05; **, *P < *0.01; ***, *P < *0.001; ****, *P < *0.0001 (using two-way ANOVA with the Tukey’s posttest; ns, not significant).

In addition, we analyzed and compared the molecular spatial profiles of RRV-infected quadriceps muscles of WT mice and MXRA8^−/−^ mice at 10 dpi. Regions of interest (ROIs) labeled with CD45 antibody and nuclear fluorescent antibodies were imaged and subjected to UV light, which cleaved the probe linker (Fig. 7A and B). Barcode oligonucleotides released in this way were captured by microfluidics and quantified via nCounter. PCA showed three distinct clusters as follows: (i) WT RRV-infected mice; (ii) MXRA8^−/−^ RRV-infected mice, and (iii) WT and MXRA8^−/−^ PBS-infected mice (Fig. 7C). The analysis revealed differences in the expression of several key genes. Notably, the expression of CD127/IL7RA, CD45, BatF3, IFNGR, Ly6G/Ly6C, CD40, CD127, F4/80, and MHC-II was determined to be higher in the RRV-infected MXRA8^−/−^ group compared to RRV-infected WT group (Fig. 7D and Fig. 8; see also Fig. S7), whereas PD-L1, ER, CD44, CD86, CD11c, CD4, CD8, fibronectin, *K_i_*-67, CD11b, and CD3 were shown to be upregulated in the RRV-infected WT group compared to the infected MXRA8^−/−^ group (Fig. 7D and Fig. 8; see also Fig. S7). Notably, genes, including GZMB, CD127/ILRA, Ly6G/Ly6C, CD14, CD31, AhR, BatF3, and CD163, were downregulated in the RRV-infected groups compared to mock-infected groups (Fig. 7D and Fig. 8; see also Fig. S7).

10.1128/mbio.00588-23.7 FIG S7 Heatmap of immune transcriptional profiles in the quadriceps RRV-infected MXRA8^−/−^ mice. WT and MXRA8^−/−^ mice were infected with RRV at 10^4^ PFU or mock infected with PBS. Quadriceps were harvested from infected mice at 10 dpi. Gene transcription profiles were analyzed using R. Differences in gene expression were plotted out in heatmap. The data are normalized to a WT control group, and differential expression is shown as the Z-score. Download FIG S7, PDF file, 0.6 MB. Copyright © 2023 Ng et al. 2023 Ng et al. https://creativecommons.org/licenses/by/4.0/ This content is distributed under the terms of the Creative Commons Attribution 4.0 International license.

## DISCUSSION

Using an established RRV mouse model, we showed that disease signs were reduced in RRV-infected MXRA8^−/−^ mice compared to WT mice. Reduced disease was associated with significantly diminished cellular infiltrates in the quadriceps muscles and ankle joints of RRV-infected MXRA8^−/−^ mice. It has been reported previously that inflammatory monocytes, macrophage-derived proinflammatory factors and the presence of cellular infiltrates contribute to acute alphavirus disease (20–23). Our flow cytometry results further highlight the role of T cells, macrophages, inflammatory monocytes, neutrophils, and NK cells in RRV disease pathology. To further characterize the leukocyte responses during RRV disease with or without MXRA8, Cytometry by Time of Flight (CyTOF) was employed to decipher the development of leukocytes and the associated cell markers in lymphoid organs of the infected mice. Interestingly, CCR7, Ly6C, and CD11b were identified to be the major factors contributing to the kinetics of phenotypic change in lymphocytes between WT and MXRA8^−/−^ mice. The differences in myeloid cells between WT and MXRA8^−/−^ mice were mainly mediated by changes in CCR2, Ly6C, CD163, and CD64. CCR7 plays a critical role in regulating the migration of lymphocytes to secondary lymphoid tissues in response to inflammation (24). Ly6C is expressed on plasma cells from the spleen and bone marrow following lipopolysaccharide activation (25). Ly6C was reported to help in the homing of central memory CD8^+^ T cells to the lymph nodes (26) and was also shown to be a surface marker for steady state of regulatory T cells (27). CD11b has been associated with various leukocyte activities, including migration, adhesion, activation, and phagocytosis, among others. In addition, CD11b was also reported to be found on memory B cells and helps to mediate homing processes (28). It was also present on active virus-specific CD8^+^ cytotoxic T cells and virus-specific memory T cell populations (29). The lymphocytes of RRV-infected MXRA8^−/−^ mice exhibited delayed and weaker upregulation of CCR7, Ly6C, and CD11b expression compared to WT mice. These findings suggest that these three molecules play a role in the regulation of the cellular immune response during the pathogenesis of RRV disease. CCR2, Ly6C, CD163, and CD64 were shown to be involved in the recruitment and activation of inflammatory monocytes and macrophages during alphavirus disease pathogenesis (23, 30). Our present study revealed that in RRV-infected MXRA8^−/−^ mice, myeloid cells expressed these surface antigens at a lower rate and at lower levels compared to those in RRV-infected WT mice. The diminished activation of these four cell markers was in line with our flow cytometry data showing reduced cell populations of monocytes and macrophages in the infected MXRA8^−/−^ mice. Further studies are needed to elucidate the interplay between decreased virus replication and the postponed/reduced activation of specific surface antigens on leukocytes and how the network and mechanisms of immune regulators control the pathogenesis of RRV disease.

In line with this altered leukocyte profile, the levels of a range of proinflammatory cytokines and chemokines were also reduced in MXRA8^−/−^ mice compared to WT mice. Notably, however, IL-17A was observed at higher levels in RRV-infected MXRA8^−/−^ mice at 3 dpi. IL-17 has recently been shown to contribute to CHIKV and RRV disease (31, 32). The upregulation of IL-17A at 3 dpi of RRVD suggests MXRA8 deficiency may alter T cell differentiation of the Th17 axis during the onset of RRVD. Interestingly, IL-17 was not detectable in CHIKV-infected MXRA8^−/−^ mice (14). The highly upregulated IL-17 in RRV-infected MXRA8^−/−^ mice demonstrates a critical difference in the pathogenesis between CHIKV and RRV.

Arthritogenic alphavirus infection has been shown to have a direct impact on skeletal health. Clear evidence of peri-articular bone loss within the tibial epiphysis and bone loss within the vertebrae of RRV-infected mice was observed in a previous study. The main contributor to this bone loss was postulated to be the increased RANKL/OPG ratio, which drives up osteoclastogenesis (18). Cartilage degradation was also highlighted in RRV disease in a recent study (19). Our results showed that the articular cartilage of RRV-infected MXRA8^−/−^ mice was less compromised compared to RRV-infected WT mice. Despite the lack of statistical significance, the articular cartilage in mock-infected MXRA8^−/−^ mice was shown to be slightly thinner compared to the mock-infected WT mice. This observation is in line with a previous study that suggested MXRA8 was required for the proliferation and maturation of chondrocytes via Indian hedgehog pathway (Ihh) (33). Ihh plays an important role in bone formation and is known to induce osteoblast differentiation in the perichondrium (34). Therefore, in the absence of MXRA8, endochondral ossification may be partially impaired leading to thinner articular cartilage thickness.

MXRA8 was also reported to inhibit osteoclast differentiation (9). We observed that the number of osteoclasts (TRAP-positive cells) was slightly higher in mock-infected MXRA8^−/−^ mice than in mock-infected WT mice, which is consistent with the findings of the previous study. Interestingly, the number of TRAP-positive cells in both RRV-infected groups was found to be equivalent despite clear evidence of compromised cartilage in the RRV-infected WT group. In addition, the bone of RRV-infected WT mice was found to be extremely brittle during the tissue collection, and the femoral heads could more easily be detached from the acetabulum, compared to RRV-infected MXRA8^−/−^ mice, which showed little to no phenotypical difference compared to the mock-infected groups. These observations indicated that although osteoclasts were found to be at a comparable level between RRV-infected WT and MXRA8^−/−^ mice at day 10 postinfection, cartilage/bone damage in RRV-infected in WT mice was more prominent compared to MXRA8^−/−^ mice. To fully characterize the role of osteoclasts in RRV-induced bone pathology, further studies are required to characterize the complete kinetics of osteoclast development in the course of (particularly the early stage) RRV disease pathogenesis.

Transcriptional studies revealed the upregulation of two genes, Cdkn1a and Ifi44, in RRV-infected MXRA8^−/−^ mice at day 7 postinfection in blood. Gene Cdkn1a encodes p21, which is known to be involved in the suppression of HIV-1 replication *in vitro*, by interacting with viral proteins to reduce virus replication (35–37). Subsequent studies conducted in human peripheral blood mononuclear cells demonstrated the protective effect of Cdkn1a gene against HIV-1 disease progression (38). Our result demonstrated an upregulation of Cdkn1a in MXRA8^−/−^ mice, which suggests a protective role of Cdkn1a against RRV in mice. Ifi44 is also shown to be upregulated in RRV-infected MXRA8^−/−^ mice compared to RRV-infected WT mice. In transcriptomic studies Ifi44 has been shown to be upregulated in CHIKV disease (39, 40). The IFN-α-inducible protein Ifi44 is a cytoplasmic protein that stimulates an antiproliferative state in cells, initially found to be involved in the forming of microtubular structures (41, 42). Notably, Ifi44 was also demonstrated to negatively modulate IFN responses and therefore supports virus replication (43). Furthermore, Hallen et al. (41) showed that Ifi44-high expression was associated with more neutrophils, NK cells, M1 macrophages, M2 macrophages, Th1 and Th2 CD4^+^ T cells, CD8^+^ effector memory T cells, and plasmacytoid dendritic cells in the tumor microenvironment. The higher level of Ifi44 gene in RRV-infected MXRA8^−/−^ mice might be a novel mechanism by which RRV modulates IFN response to facilitate replication in the absence of the entry receptor (44, 45). The role of Cdkn1a and Ifi44 in alphavirus disease warrants further study.

We leveraged the GeoMX platform to compare spatial profiles in immune-rich regions of the quadriceps in infected and uninfected MXRA8^−/−^ and WT mice. The genes of PD-L1, ER, CD44, CD86, CD11c, CD4, CD8, fibronectin, Ki-67, CD11b, and CD3 were shown to be upregulated in the RRV-infected WT group compared to the infected MXRA8^−/−^ group. However, it is interesting that the expression of CD127/IL7RA, CD45, BatF3, IFNGR, Ly6G/Ly6C, CD40, CD127, F4/80, and MHC-II were higher upregulated in RRV-infected MXRA8^−/−^ mice. These genes are known to be prominently involved in virus infection-associated inflammation. Considering the diminished inflammation in RRV-infected MXRA8^−/−^ mice, elevation of these genes raises questions of how these genes are upregulated under mild inflammatory conditions and to what effect and whether these changes are virus- or host-driven. One possible explanation for this seemingly paradoxical upregulation of these genes could be that it is a compensatory effect for the lower total CD45^+^ infiltrate in RRV infection. Another plausible explanation might be attributed to leukocyte trafficking. In the RRV-infected WT mice, higher levels of proinflammatory cytokines/chemokines were detected, which attracted more leukocyte infiltration into the ROIs, compared to MXRA8^−/−^ mice. Notably, the component of the infiltrated leukocytes might be different between the infected WT and MXRA8^−/−^ mice. If the expression level of genes of interest is normalized against populations with various components, it could result in differences in their expression levels.

The antibody-dependent enhancement (ADE) pathway has been identified during RRV infection in mouse macrophages (46, 47). Interestingly, in MXRA8^−/−^ mice, low levels of RRV replication were observed in the present and previous studies (14), indicating other entry mechanisms were employed by RRV during infection. We therefore hypothesize that, through Fc receptor-mediated ADE, RRV is likely to infect macrophages in MXRA8^−/−^ mice. This hypothesis warrants future investigation.

In summary, our work provides new insight on the role played by MXRA8 in alphavirus-induced diseases. Due to the lack of crucial cellular entry receptor, the viral load in the tissues of MXRA8^−/−^ mice was diminished. The cellular immune response and levels of proinflammatory cytokines in these tissues were therefore significantly lower compared to WT mice in which RRV replicated to a higher level. As a result, ameliorated skeletal muscle and cartilage/bone destruction were observed in MXRA8^−/−^ mice. More importantly, we have shown in this study that MXRA8 clearly plays more than a receptor role in the pathogenesis of alphavirus-induced disease. Specifically, while the viral load and disease symptoms decreased, NanoString analysis revealed higher levels of cell surface antigens and genes in leukocytes of RRV-infected MXRA8^−/−^ mice compared to WT mice, suggesting potential novel mechanisms by which RRV facilitates its replication. Furthermore, CyTOF analysis revealed a range of leukocyte surface markers that exhibited a significant delay in upregulation in the infected MXRA8^−/−^ mice, which represents a novel observation in RRV-induced inflammation in animals lacking MXRA8. The differences in the kinetics of cell marker expression between the WT and MXRA8^−/−^ mice suggest that MXRA8, in addition to facilitating virus entry, may also regulate the cellular immune response.

## MATERIALS AND METHODS

### Ethics.

All experiments were performed in accordance with the guidelines set out by the Griffith University Animal Ethics Committee for Ethics in Animal Experiments (MHIQ/01/19). The generation of MXRA8^−/−^ mice used in this study was supported by Phenomics Australia and the Australian Government through the National Collaborative Research Infrastructure Strategy (NCRIS) program.

### Virus.

Virus stocks of RRV-T48 strain from the full-length T48 cDNA clone were generated through the *in vitro* transcription of SacI-linearized plasmid pRR64 using SP6-specific mMessage mMachine *in vitro* transcription kits (Ambion), kindly provided by Richard Kuhn, Purdue University, West Lafayette, IN. Briefly, 2 μg of RRV-T48 cDNA plasmids were linearized, transcribed, and electroporated into Vero cells using an electroporator (Eppendorf). Recovered viruses were amplified in Vero cells. The virus titers were determined by using a standard plaque-forming assay on Vero cells. The virus stocks were stored in 0.5-mL aliquots at −80°C.

### Generation of MXRA8 knockout mice.

MXRA8^−/−^ mice was made by injecting 20 ng/μL of Cas9 mRNA and 10 ng/μL of sgRNA (GCTATCCATCGTGACGACTC and GCCACGAGGGACTTTGCATT) into the cytoplasm of fertilized one-cell stage embryos generated from WT C57BL/6J breeders. After 24 h, two-cell stage embryos were transferred into the uteri of pseudopregnant female mice. Viable offspring were genotyped by next-generation sequencing and bred in-house to establish germ line homozygosity. These MXRA8^−/−^ mice do not show any defects in development and fertility (see Fig. S1).

10.1128/mbio.00588-23.1 FIG S1 Generation of MXRA8 knockout mice. (A) MXRA8^−/−^ knockout mice were made by injecting 20 ng/μL of Cas9 mRNA and 10 ng/μL of the sgRNAs, respectively, into the cytoplasm of fertilized one-cell stage embryos generated from WT C57BL/6J breeders. After 24 h, two-cell stage embryos were transferred into the uteri of pseudopregnant female mice. Viable offspring were genotyped by next-generation sequencing. (B) Representative gel of MXRA8 genotyping. The expected bands are 326 bp for deleted allele and 371 bp for WT allele. (C) Next-generation sequencing of F_0_ offspring. InDel mutations are highlighted in yellow. (D) Establishment of germ line homozygosity. F_0_ mosaic mice were bred with WT mice to generate heterozygous MXRA8^+/–^ mice. MXRA8^+/–^ mice were bred in pairs to generate MXRA8^−/−^ mice. Download FIG S1, PDF file, 2.4 MB. Copyright © 2023 Ng et al. 2023 Ng et al. https://creativecommons.org/licenses/by/4.0/ This content is distributed under the terms of the Creative Commons Attribution 4.0 International license.

### Mouse infections and disease monitoring.

C57BL/6J WT mice were obtained from the Animal Resource Centre (ARC; Australia) whenever necessary. MXRA8^−/−^ mice were generated by the MAGEC laboratory (WEHI) on a C57BL/6J background and bred in-house.

C57BL/6J and MXRA8^−/−^ mice were inoculated subcutaneously in the thorax below the right forelimb with RRV diluted to 10^4^ PFU to a final volume of 50 μL in PBS at 20 to 22 days old. Mock-infected mice were inoculated similarly with PBS only.

Mice were monitored daily, and clinical scores and weights were recorded until euthanizing time points. Clinical symptoms were scored as follows: 0, no disease signs; 1, ruffled fur; 2, mild hind limb weakness; 3, moderate hind limb weakness; 4, severe hind limb weakness and dragging of hind limbs; 5, complete loss of hind limb function; and 6, moribund. Mice showing weight loss greater than 15% from previous day with a clinical score of 6 were euthanized via asphyxiation.

### Plaque assay.

Vero cells were grown to a density of 2 × 10^5^ cells per well in 12-well plates and incubated overnight at 37°C in a humidified CO_2_ incubator. Culture medium was aspirated using a vacuum pump and washed once with sterile PBS. Cells were incubated with virus overlay for 1 h at 37°C in a humidified CO_2_ incubator. An agarose overlay was added to the well, followed by incubation in a humidified CO_2_ incubator. The agarose overlay was aspirated from wells using a vacuum pump, and the cell monolayer was stained with crystal violet. Plaques were calculated using the following formula: PFU/mL = *n*/(*D* × *V*), where *n* is the number of plaques counted, *D* is the dilution factor, and *V* is the volume of diluted virus in mL added to well.

### Histology.

Mouse were culled at experiment endpoint. Quadriceps and joint tissues were harvested and transferred into Falcon tubes containing 4% paraformaldehyde (PFA) and fixed on a rotator at 4°C. Quadriceps tissues were washed with PBS for 1 h the next day and stored at 70% ethanol (EtOH) for paraffin infiltration. Ankle tissues were decalcified in 14% EDTA in for 10 days (the solution was changed every 2 days) after 48 h of PFA fixation and stored at 70% EtOH for paraffin infiltration. Tissues were penetrated with paraffin using the Leica TP1020 tissue processor and embedded using Myr Tissue Embedding Centre EC 500 into paraffin blocks.

Paraffin blocks containing tissue samples were sectioned using a Leica RM2245 semiautomated rotary microtome into 5-μm-thick slices and transferred onto a water bath set at 38°C to unfurl wrinkled sections. Unfurled sections were collected using positive charged slides. Slides were dried overnight before subjected to deparaffinization and rehydration. Rehydrated slides were then stained with H&E, safranin-O, and TRAP according to the manufacturer’s protocol. Stained slides were imaged with a Leica Aperio AT2 and exported to QuPath analysis software to quantify the cellular infiltrates and analyze tissue damage. The numbers of TRAP-positive cells were analyzed using the ImageJ Cell Counter plugin.

### Flow cytometry.

Mouse quadriceps and spleen were harvested into weighted 2-mL Eppendorf tubes containing 1 mL of RP10 with collagenase IV for quadriceps and 10-mL tubes containing 4 mL of RP10 for spleen. For quadriceps, the tubes were then weighed before tissues were minced into slurry and incubated at a 37°C water bath for 1.5 h. The slurries were transferred into a 10-mL tube added with 3 mL of RP10 and pipetted vigorously. Cell lysates were filtered through a 70-μm-pore-size cell strainer into a fluorescence-activated cell sorting (FACS) tube and centrifuged at 400 × *g* for 5 min at 4°C. Supernatants were decanted, and cell pellets were resuspended with 1 mL of FACS buffer. Cell lysates were filtered through a 30-μm-pore-size cell strainer into a FACS tube and centrifuged at 400 × *g* for 5 min at 4°C. The supernatants were decanted, and cell pellets were resuspended with 200 μL of FACS buffer.

For spleen, tubes were weighted and filtered through a 70-μm-pore-size cell strainer into a 50-mL Falcon tube. The tubes were centrifuged at 400 × *g* for 5 min at 4°C. The supernatants were decanted, and cells were resuspended in 1 mL of ACK lysis buffer and then left on ice for 2 min. The tubes were topped up with 5 mL of FACS buffer and centrifuged at 400 × *g* for 5 min at 4°C. The supernatants were decanted, and cell pellets were resuspended with 8 mL of FACS buffer. Then, 200 μL was taken from the 8 mL of cell suspension, filtered through a 30-μm-pore-size cell strainer into a FACS tube, and centrifuged at 400 × *g* for 5 min at 4°C. The supernatants were discarded, and the cells were resuspended in 500 μL of FACS buffer.

The cells were resuspended in staining buffer (PBS with 2% fetal calf serum and 5 mM EDTA), blocked with anti-CD16/32 Fc block (BD Biosciences, Franklin Lakes, NJ), and labeled with fluorochrome antibodies (CD45 [HI30; BD Biosciences], CD3 [145-2C11; Invitrogen], CD4 [RM4-5; BioLegend], CD8 [53-6.7; BioLegend], Ly6C [HK1.4; BioLegend], Ly6G [1A8; BD Biosciences], CD11b [M1/70; BioLegend], and NK1.1 [PK136; BD Biosciences]). NIR (near infrared) Live/Dead stain (Life Technology) was used to exclude dead cells. Counting beads (Spherobeads; BD Biosciences) were added to the samples before acquisition. Samples were acquired using a BD LSR Fortessa flow cytometer, and data analysis was performed using FlowJo 10.7.

### CyTOF.

One million mouse splenocytes and popliteal DLN cells per sample were barcoded with anti-CD45 antibodies conjugated to various metal tags, followed by incubation in FACS buffer containing 5 μg/mL anti-CD16/32 Fc block for 30 min at 4°C. Then, they were washed with 2 mL of FACS buffer and pelleted at 400 × *g* for 5 min at 4°C. CD45-barcoded samples were resuspended in 1 mL of FACS buffer and combined for subsequent labeling. The cells were then stained with 200 μL of 5 μM cisplatin solution in PBS for 5 min at room temperature, before quenching with 2 mL of FACS buffer and centrifugation at 400 × *g* for 5 min at 4°C. The supernatant was discarded, and the cells were labeled with metal-tagged antibodies (see Table S1) against surface markers in FACS buffer, followed by incubation for 30 min at 4°C. The cells were then washed with 3 mL of FACS buffer and centrifuged at 400 × *g* for 5 min at 4°C, and the supernatant was discarded. The cells were then incubated with secondary metal-tagged antibodies against the appropriate fluorophores in FACS buffer for 30 min at 4°C before they were washed with 3 mL of FACS buffer and centrifuged at 400 × *g* for 5 min at 4°C. For intracellular staining, the cells were fixed, permeabilized, and washed with an eBioscience Foxp3/transcription factor staining buffer set according to the manufacturer’s protocols, followed by incubation with metal-tagged intracellular antibodies for 30 min at 4°C. The cells were then washed with wash buffer, pelleted at 900 × *g* for 10 min, and subsequently stained with 500 μL of iridium DNA intercalator in 4% paraformaldehyde for 20 min at room temperature. Finally, the cell samples were washed in FACS buffer, followed by ultrapure water, and resuspended in Maxpar cell acquisition solution (Fluidigm) before sample acquisition with a Fluidigm Helios mass cytometer.

10.1128/mbio.00588-23.8 TABLE S1 Antibody labels for mass cytometry. Download Table S1, DOCX file, 0.02 MB. Copyright © 2023 Ng et al. 2023 Ng et al. https://creativecommons.org/licenses/by/4.0/ This content is distributed under the terms of the Creative Commons Attribution 4.0 International license.

Analysis of acquired mass cytometry data were undertaken using FlowJo v10.8 with the UMAP plugin v3.1. Sample data were imported into FlowJo, cells were identified by labeled DNA content, and doublets and dead cells were removed according to cisplatin labeling, and individual samples were demultiplexed and identified according to CD45 barcode channels. Samples from each tissue type were then concatenated, and computational analysis was performed using UMAP in FlowJo for dimensionality reduction and cell phenotype visualization. Cell type clusters were identified on the UMAP output using traditional immune cell markers. Statistical analysis of the fold change in expression of various markers was performed using GraphPad Prism v9.4.

### NanoString nCounter gene expression.

Blood was collected from RRV-infected MXRA8^−/−^ and WT mice and transferred into a PAXgene tube at a 2.8× volume of PAXgene blood reagent to 1 volume of blood (e.g., 200 to 560 μL). Total RNA extraction was performed using TRIzol reagent (Life Technologies, Australia) according to the manufacturer’s protocol. Total RNA was quantified by using a NanoDrop 1000 spectrophotometer (Thermo Scientific, Victoria, Australia). Total RNA was cleaned and concentrated using RNA Clean & Concentrator-25 (Zymo Research) according to the manufacturer’s protocol and was diluted to a concentration of 10,000 cells/mL. RNA target molecules were quantified using a nCounter mouse PanCancer immune profiling panel, and samples were processed according to the nCounter gene expression protocol.

### NanoString GeoMX DSP.

A slide mounted formalin fixed paraffin embedded section of quadricep muscle was utilized for spatial profiling using the NanoString GeoMX Digital Spatial Profiler, a custom system with integrated software. Two fluorescent markers, including CD45 and syto13 (DNA), were applied to the tissue section, and images were assembled at ×20 resolution. From these images, a total of 48 regions of interest (ROIs) from four slides were chosen for spatial analysis. Samples were subjected to antigen retrieval and stained with a cocktail of antibodies labeled with photocleavable DNA-indexing oligonucleotides to generate quantitative protein profiles. ROIs were exposed to UV light (365 nm) to release oligonucleotides, which were then captured via microfluidics and stored in the individual wells of a microtiter plate. After collection from all ROIs, oligonucleotides were hybridized to unique four-color, six-spot optical barcodes with targets enumerated on the NanoString digital analyzer.

### Cytokine and chemokine analysis.

Mice were inoculated subcutaneously with 10^4^ PFU of RRV under the right armpit, and quadriceps muscles were harvested at 3 and 7 dpi into T-PER tissue protein extraction reagent (Thermo Scientific) supplemented with 1× Halt protease inhibitor cocktail. Samples were homogenized and centrifuged at 10,000 × *g* for 5 min to pellet the tissue debris. The supernatants were collected into a new 1.5-mL tube and analyzed for cytokine and chemokine levels using a Bio-Plex mouse cytokine 23-plex assay kit (Bio-Rad, catalog no. M60009RDPD) according to the manufacturer’s instructions.

### Statistical analysis.

All required statistical approaches were applied for data comparison whenever needed using GraphPad Prism (version 9). The statistical significance for the viral titer analysis of mouse specimens, histological analysis, and flow cytometry analysis was assessed using a Mann-Whitney U test. The statistical significance for mouse disease and weight was assessed by using two-way analysis of variance (ANOVA) with a Bonferroni posttest. The statistical significance for the multiplex protein analysis of RRV-infected quadriceps homogenates was analyzed by using two-way ANOVA with a Holm-Šídák posttest. Mouse serum for NanoString nCounter was analyzed by using two-way ANOVA with a Šídák posttest. DSP NanoString analysis of mouse quadriceps samples were performed using two-way ANOVA with Tukey’s posttest.

### Data availability.

The data that support the findings of this study are available from the corresponding authors upon request.
