# Supplementary figures and images for: Role of MXRA8 in Ross River Virus Disease Pathogenesis
Source: mBio. 2023 Apr 10;14(2):e00588-23. doi: 10.1128/mbio.00588-23 (PMC10128017; doi:10.1128/mbio.00588-23)

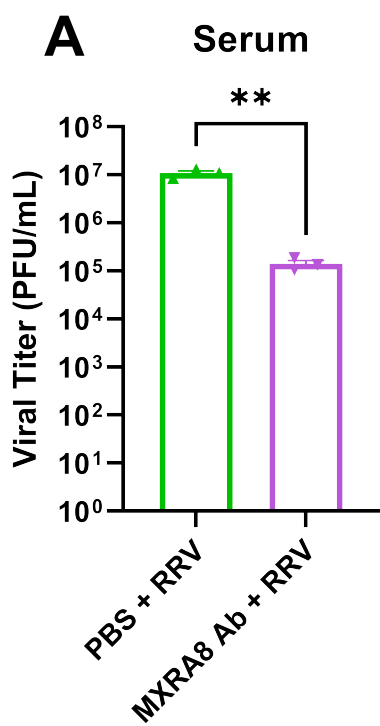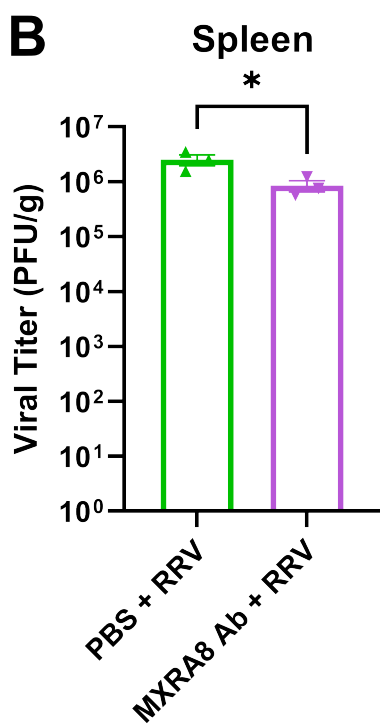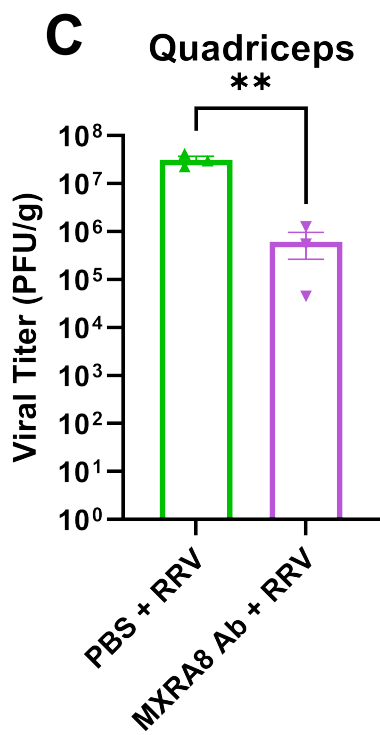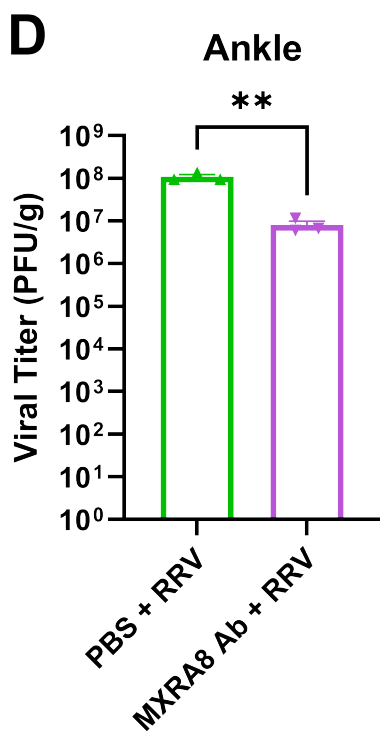

Supplement: FIG S2 [file mbio.00588-23-s0002.pdf]

# Day 10 Ankles

## A WT PBS

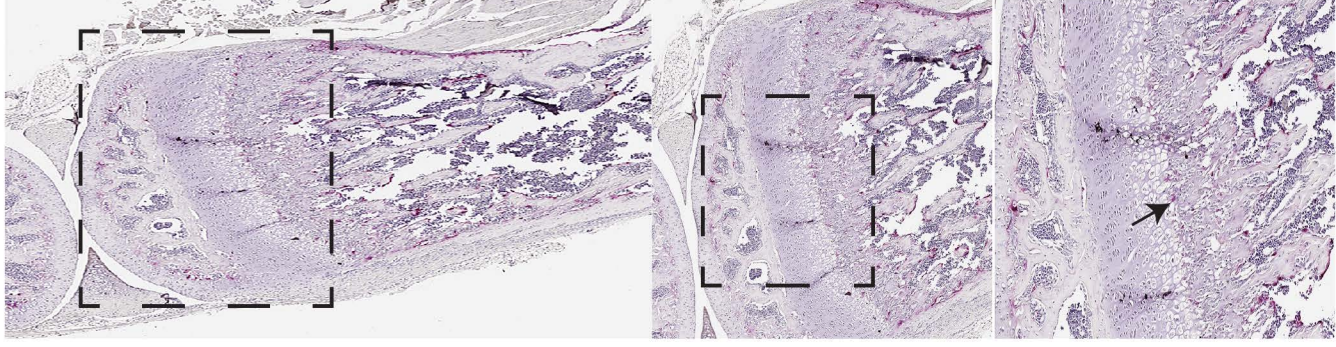

## B MXRA8<sup>-/-</sup> PBS

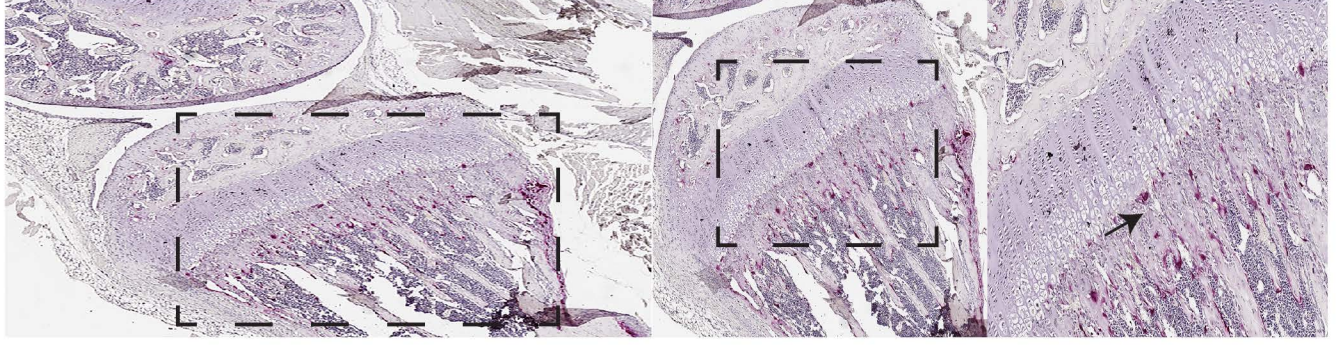

## C WT RRV

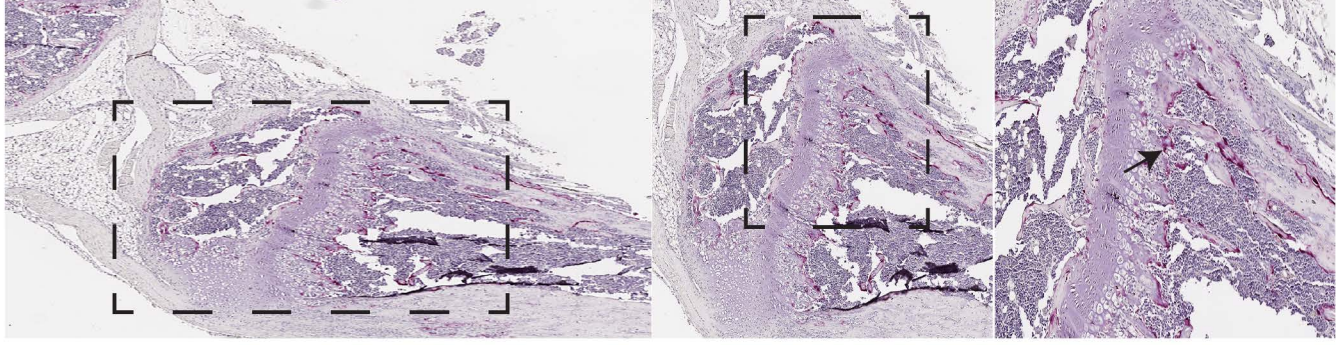

## D MXRA8<sup>-/-</sup> RRV

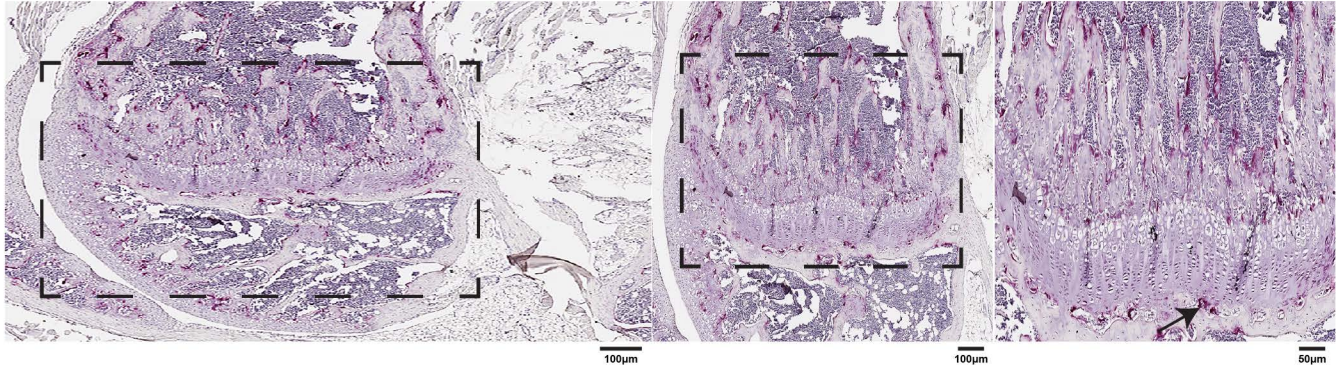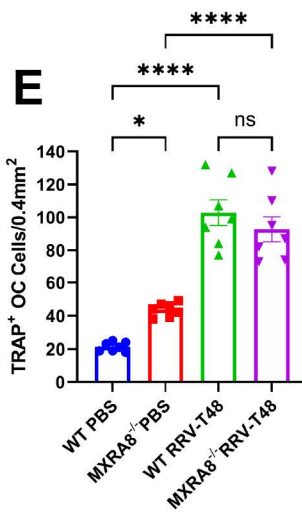

Supplement: FIG S3 [file mbio.00588-23-s0003.pdf]

DLN - Lymphocytes

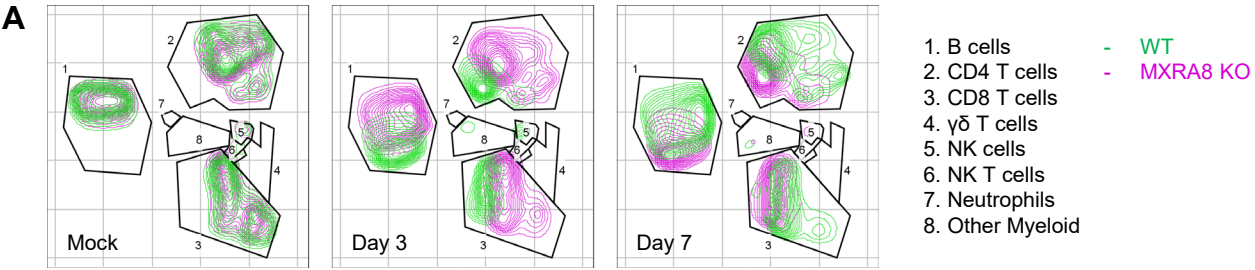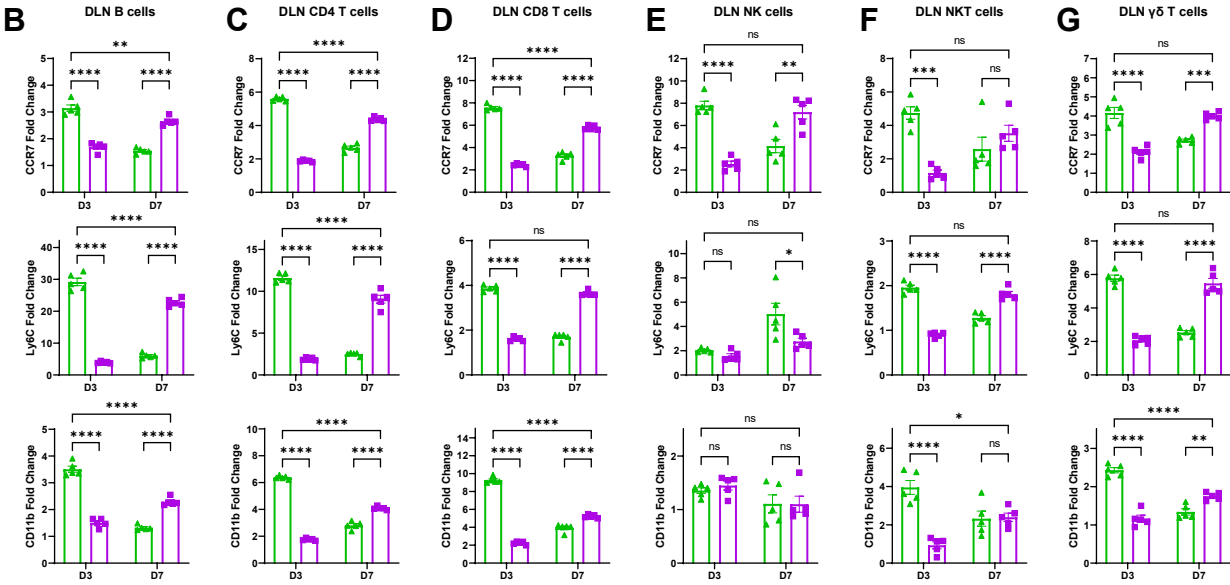

DLN - Myeloid Cells

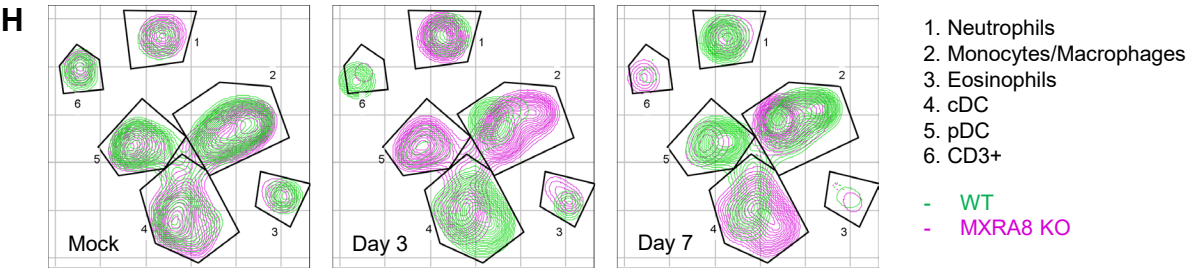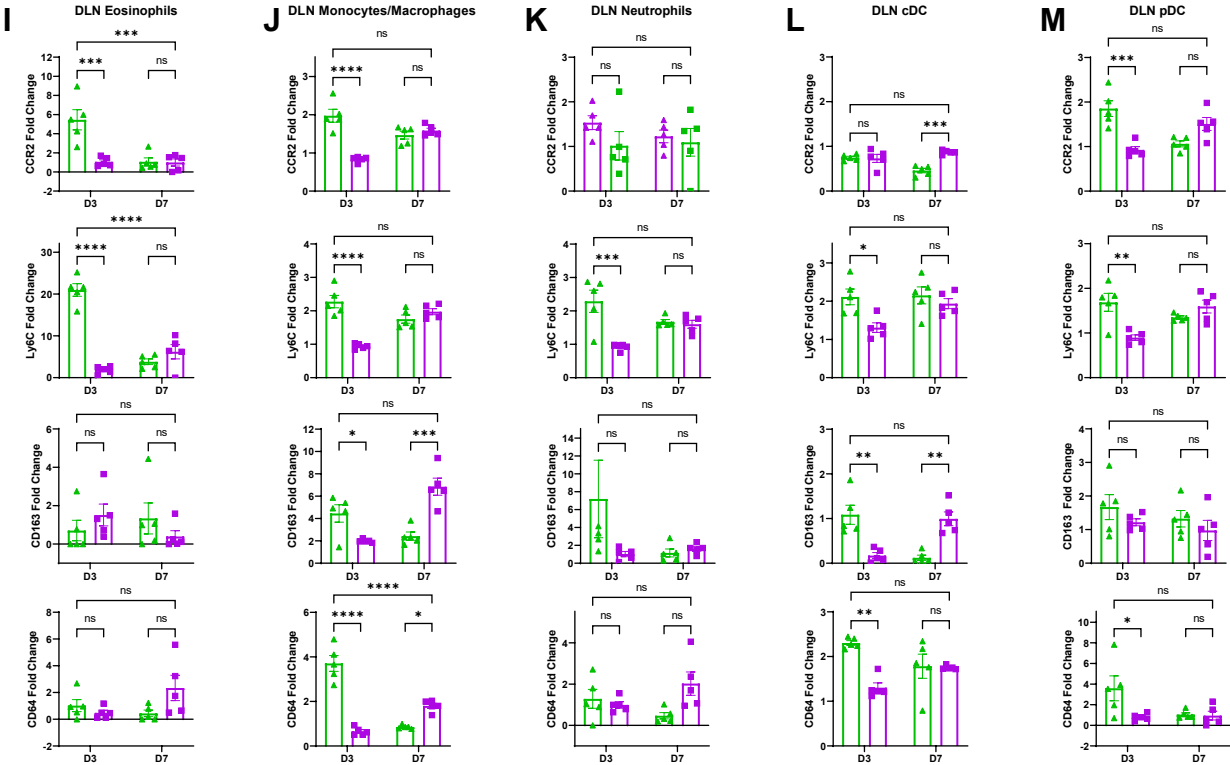

Supplement: FIG S4 [file mbio.00588-23-s0004.pdf]

**A** DLN Cell Subsets

- WT  
- MXRA8 KO

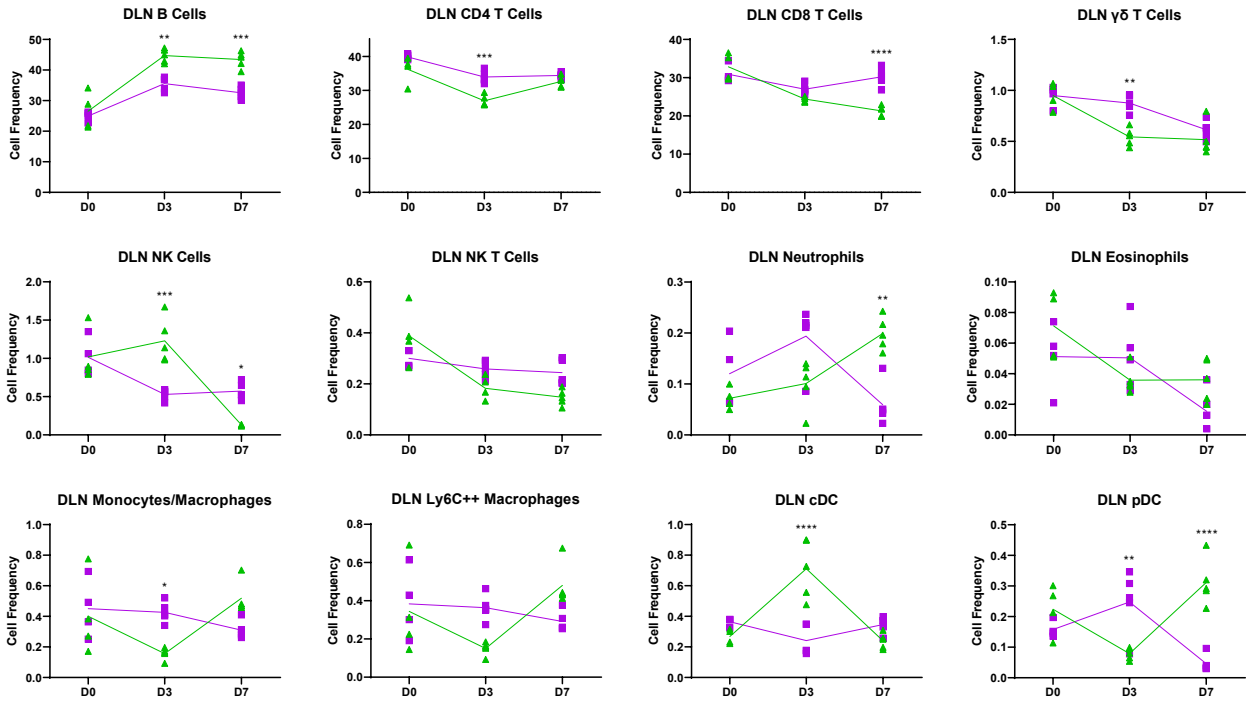

**B** Splenocyte Subsets

- WT  
- MXRA8 KO

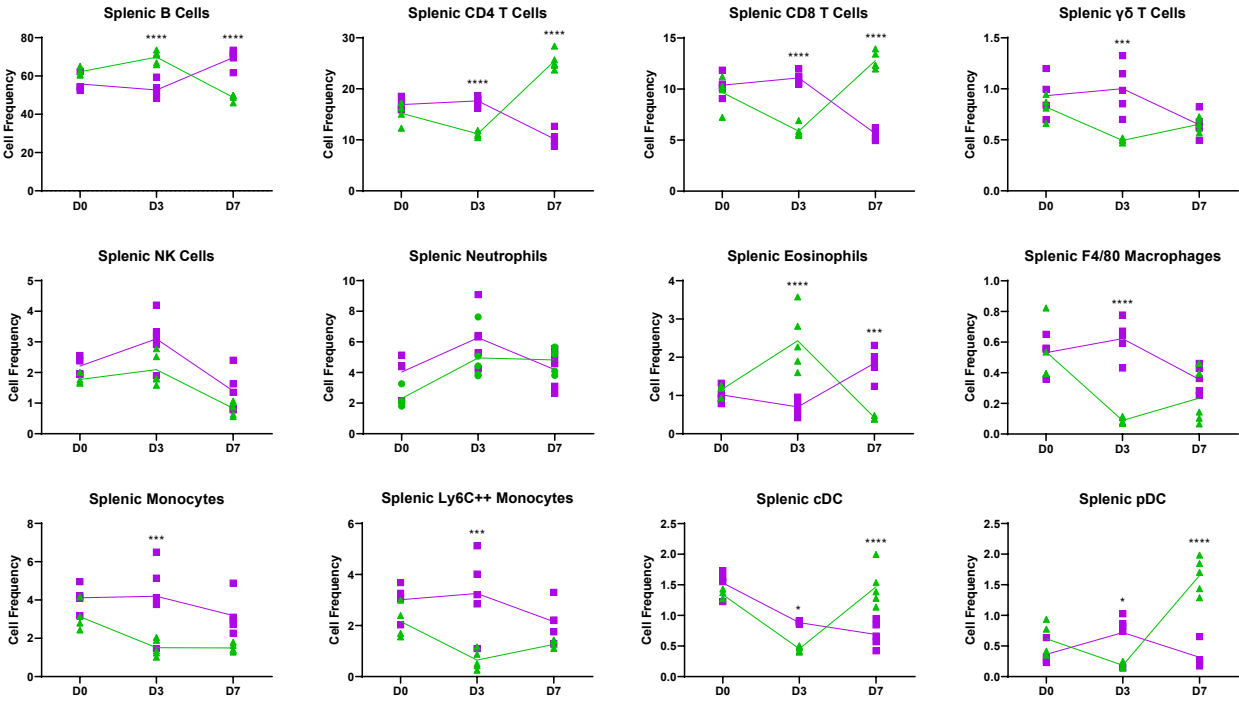

Supplement: FIG S6 [file mbio.00588-23-s0006.pdf]

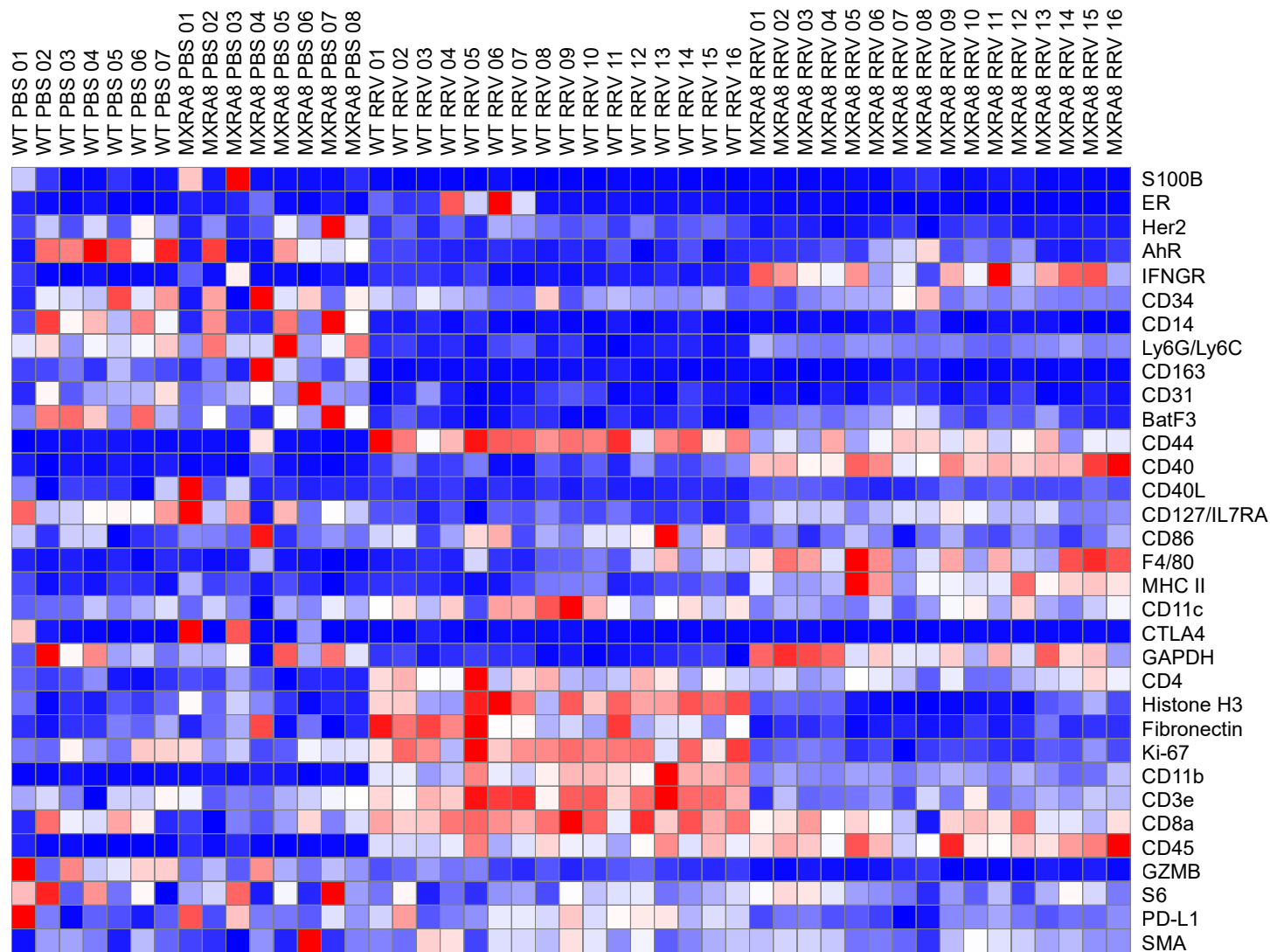

1.50

0

1.50

Supplement: FIG S7 [file mbio.00588-23-s0007.pdf]
