# Supplementary material for: Role of MXRA8 in Ross River Virus Disease Pathogenesis
Source: mBio. 2023 Apr 10;14(2):e00588-23. doi: 10.1128/mbio.00588-23 (PMC10128017; doi:10.1128/mbio.00588-23)
Supplement: FIG S5 [file mbio.00588-23-s0005.pdf]

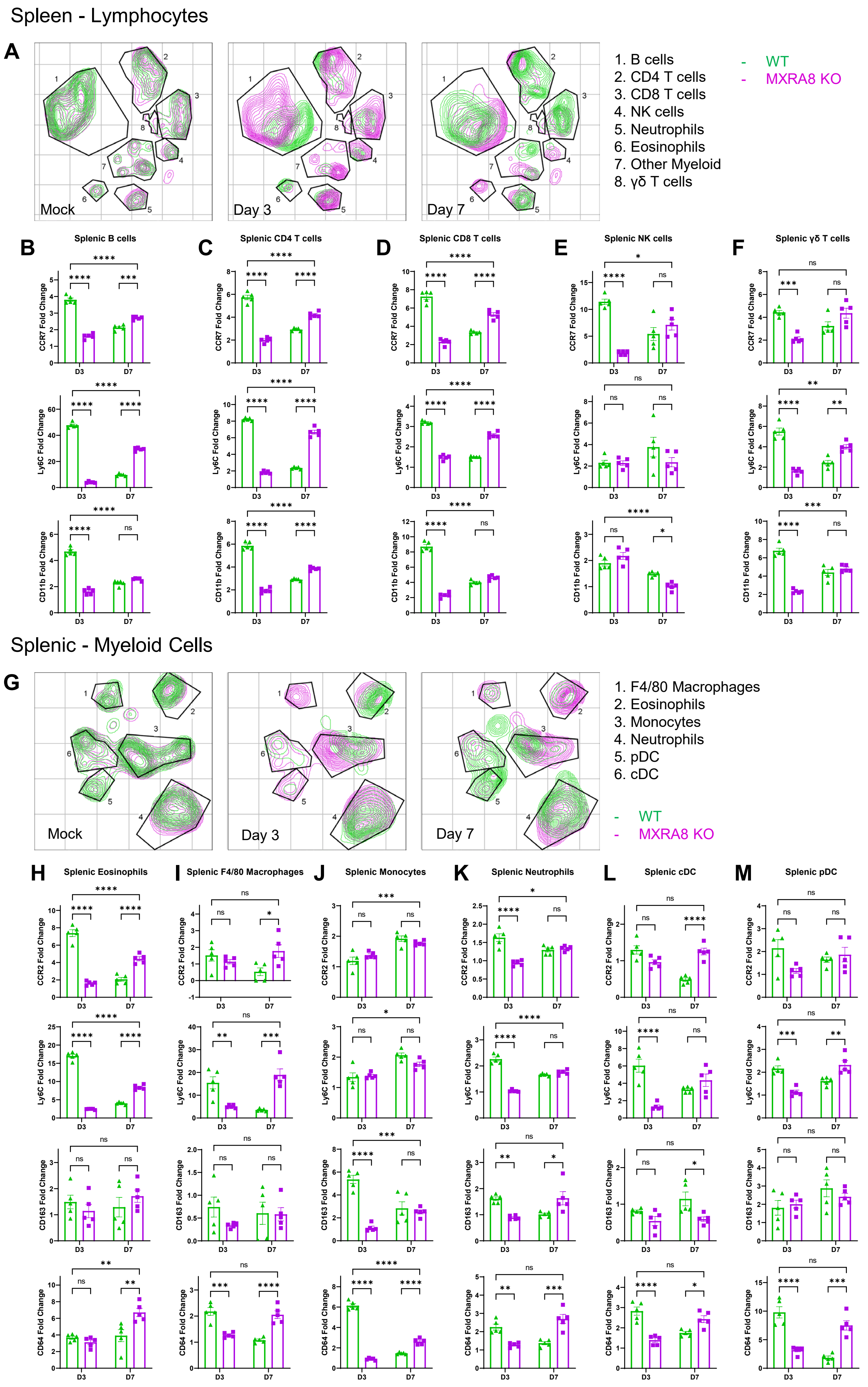
Spleen - Lymphocytes
A
Mock
Day 3
Day 7
1. B cells
2. CD4 T cells
3. CD8 T cells
4. NK cells
5. Neutrophils
6. Eosinophils
7. Other Myeloid
8. γδ T cells
- WT
- MXRA8 KO
B Splenic B cells
C Splenic CD4 T cells
D Splenic CD8 T cells
E Splenic NK cells
F Splenic γδ T cells
CCR7 Fold Change
Ly6C Fold Change
CD11b Fold Change
D3
D7
Splenic - Myeloid Cells
G
1. F4/80 Macrophages
2. Eosinophils
3. Monocytes
4. Neutrophils
5. pDC
6. cDC
- WT
- MXRA8 KO
H Splenic Eosinophils
I Splenic F4/80 Macrophages
J Splenic Monocytes
K Splenic Neutrophils
L Splenic cDC
M Splenic pDC
CCR2 Fold Change
Ly6C Fold Change
CD163 Fold Change
CD64 Fold Change
