# Supplementary material for: Role of MXRA8 in Ross River Virus Disease Pathogenesis
Source: mBio. 2023 Apr 10;14(2):e00588-23. doi: 10.1128/mbio.00588-23 (PMC10128017; doi:10.1128/mbio.00588-23)
Supplement: FIG S1 [file mbio.00588-23-s0001.pdf]

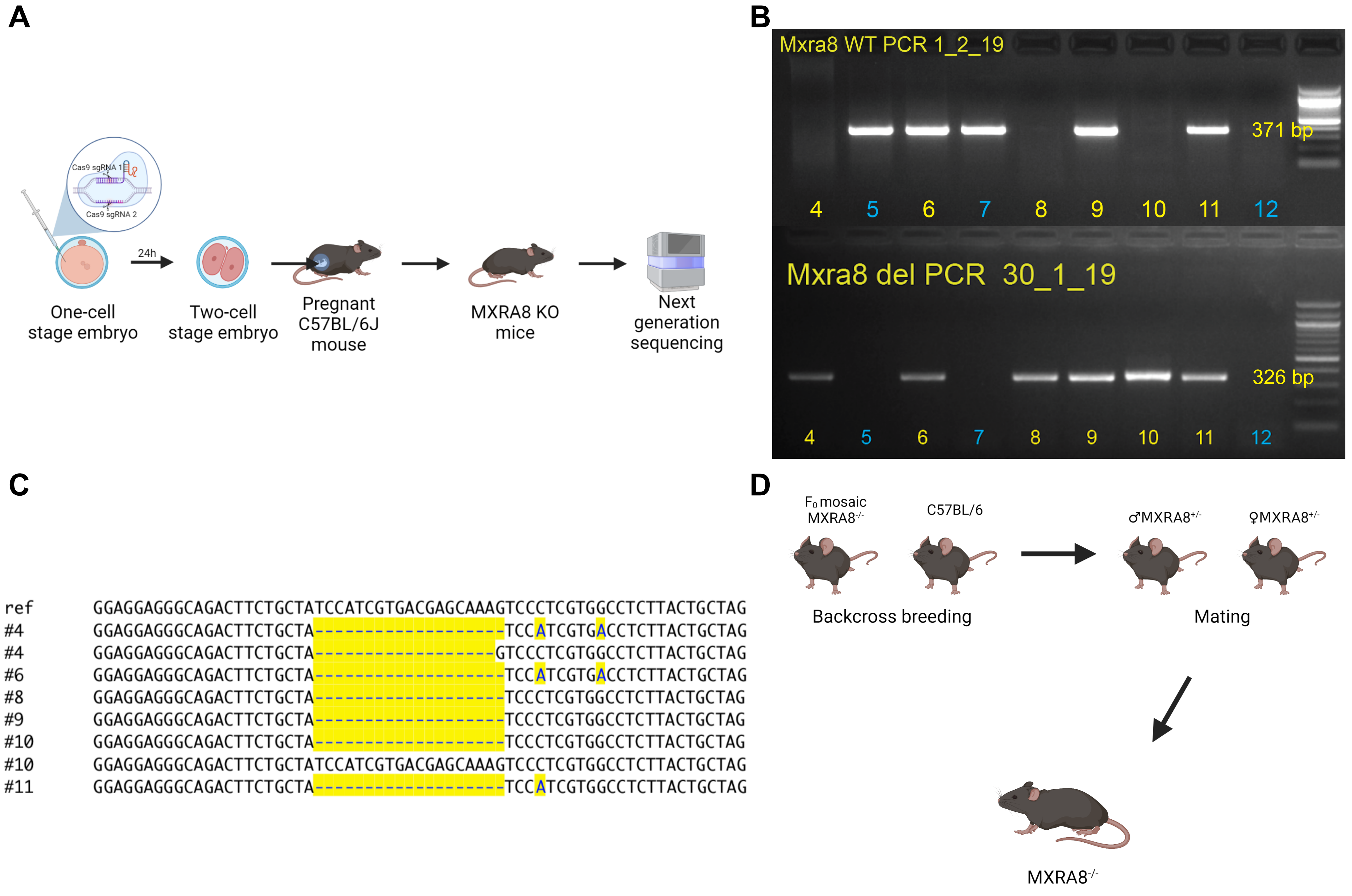

A
Cas9 sgRNA 1
Cas9 sgRNA 2
24h
One-cell stage embryo
Two-cell stage embryo
Pregnant C57BL/6J mouse
MXRA8 KO mice
Next generation sequencing
B
Mxra8 WT PCR 1_2_19
371 bp
4 5 6 7 8 9 10 11 12
Mxra8 del PCR 30_1_19
326 bp
4 5 6 7 8 9 10 11 12
C
ref GGAGGAGGGCAGACTTCTGCTATCCATCGTGACGAGCAAAGTCCCTCGTGGCCTCTTACTGCTAG
#4 GGAGGAGGGCAGACTTCTGCTA-------------------TCCATCGTGACCTCTTACTGCTAG
#4 GGAGGAGGGCAGACTTCTGCTA------------------GTCCCTCGTGGCCTCTTACTGCTAG
#6 GGAGGAGGGCAGACTTCTGCTA-------------------TCCATCGTGACCTCTTACTGCTAG
#8 GGAGGAGGGCAGACTTCTGCTA-------------------TCCCTCGTGGCCTCTTACTGCTAG
#9 GGAGGAGGGCAGACTTCTGCTA-------------------TCCCTCGTGGCCTCTTACTGCTAG
#10 GGAGGAGGGCAGACTTCTGCTA-------------------TCCCTCGTGGCCTCTTACTGCTAG
#10 GGAGGAGGGCAGACTTCTGCTATCCATCGTGACGAGCAAAGTCCCTCGTGGCCTCTTACTGCTAG
#11 GGAGGAGGGCAGACTTCTGCTA-------------------TCCATCGTGGCCTCTTACTGCTAG
D
F_0 mosaic MXRA8$^{-/-}$
C57BL/6
♂MXRA8$^{+/-}$
♀MXRA8$^{+/-}$
Backcross breeding
Mating
MXRA8$^{-/-}$
