# Supplementary material for: Role of MXRA8 in Ross River Virus Disease Pathogenesis
Source: mBio. 2023 Apr 10;14(2):e00588-23. doi: 10.1128/mbio.00588-23 (PMC10128017; doi:10.1128/mbio.00588-23)
Supplement: TABLE S1 [file mbio.00588-23-s0008.docx]

**Supplementary Table 1. Antibody labels for mass cytometry.**

| Specificity | Clone # | Conjugated Mass Label |
| --- | --- | --- |
| CD16/32 | 2.4G2 | Unlabelled (Fc Block) |
| B220 | RA3-6B2 | 89Y-B220 |
| CD45 | 30-F11 | 104Pd-CD45 |
| CD45 | 30-F11 | 106Pd-CD45 |
| CD45 | 30-F11 | 108Pd-CD45 |
| CD45 | 30-F11 | 110Pd-CD45 |
| CD69 | H1.2F3 | 139La-CD69 |
| Ly6G | 1A8 | 141Pr-Ly6G |
| CD11c | N418 | 142Nd-CD11c |
| CD194 | 2G12 | 143Nd-CD194 |
| Siglec-F | E50-2440 | 146Nd-Siglec-F |
| CD11b | M1/70 | 148Nd-CD11b |
| CD80 | 16-10A1 | 149Sm-CD80 |
| CD103 | 2E7 | 150Nd-CD103 |
| F4/80 | BM8 | 151Eu-F4/80 |
| CD4 | RM4-5 | 153Eu-CD4 |
| CD163 | S15049I | 154Gd-CD163 |
| CD138 | 281-2 | 155Gd-CD138 |
| CD48 | HM48-1 | 156Gd-CD48 |
| FOXP3 | FJK-16s | 158Gd-FoxP3 |
| CD117 | 2B8 | 159Tb-CD117 |
| CD62L | MEL-14 | 160Gd-CD62L |
| CD25 | 3C7 | 161Dy-CD25 |
| Ki67 | 11F6 | 162Er-Ki67 |
| CD197 | 4B12 | 163Dy-CD197 |
| Ly6A/E | D7 | 164Er-Ly6CA/E |
| CD115 | AFS98 | 165Ho-CD115 |
| CD19 | 6D5 | 166Er-CD19 |
| CD185 | 2G8 | 167Er-CD185 |
| CD8a | 53-6.7 | 168Er-CD8a |
| TCRγδ | GL3 | 169Tm-TCRgd |
| NK1.1 | PK136 (B6) | 170Er-NK1.1 |
| CD44 | IM7 | 171Yb-CD44 |
| CD127 | A7R34 | 173Yb-CD127 |
| MHC-II IA/IE | M5/114.15.2 | 174Yb-MHCII-IA-IE |
| CD192 | 475301 | 175Lu-CCR2 |
| Ly6C | HK1.4 | 176Lu-Ly6C |
| DNA label | N/A | 191/193Ir |
| Cisplatin (Live/dead) | N/A | 194/195Pt |
| CD3ε | 145-2C11 | 209Bi-CD3 |
